# Fear, stigma and Uncertainty: The short and long-term effects of Ebola on survivors, affected families, and community in Bundibugyo, Western Uganda

**DOI:** 10.1371/journal.pone.0355778

**Published:** 2026-09-08

**Authors:** Shamilah Namusisi, Jacinta Mukulu Waila, Sarah J. Hoffman, Cheryl Roberston, Katey Pelican, Michael Mahero

**Affiliations:** 1 Makerere University, Kampala, Uganda; 2 Heidelberg Institute of Global Health, University of Heidelberg, Germany; 3 School of Nursing, University of Minnesota, United States of America; 4 Director, Health Sciences Institute, University of Hawaii, United States of America; 5 University of Tennessee College of Veterinary Medicine, Department of Veterinary Population Medicine, University of Minnesota, Saint Paul, United States of America; Virginia Commonwealth University, UNITED STATES OF AMERICA

## Abstract

In 2014, Uganda was identified as a high-risk country for Ebola Virus Disease (EVD), with a series of outbreaks recorded since 2000. In 2007, the second outbreak in Bundibugyo district resulted in 149 reported cases and 37 confirmed Ebola deaths. Through the outbreak response, a new strain of the Ebola virus (*Bundibugyo ebolavirus)* was discovered. Although much is known about the nature of Ebola, including disease signs and symptoms, transmission and management, there is limited understanding of the short and long-term sociocultural impacts of the disease in communities. The study team conducted a focused ethnography in Bundibugyo District 10 years after the 2007 outbreak. Data collection included a review of archival data, participant observation, field notes and 19 in-depth interviews with survivors and affected families. Results underscored short and long term social, cultural and economic disruptions caused by the outbreak. We interpreted findings through an Eco-health framework with an emphasis on ways that underlying stigma accentuated detrimental long-term effects of the outbreak. Affected women, particularly widows, experienced social exclusion, and economic strain, and acknowledged loss of opportunity for a better life for their orphaned children. Deepening fear of the possible recurrence of Ebola resulted in ethnic tension driven by speculations on the 2007 outbreak source. Survivors reported varying persistent health effects including impaired vision and general body weakness. Community members reported positive changes in health seeking behaviors. Health care workers described high levels of alert for early clinical signs of Ebola, a critical factor for early outbreak detection at the community level. Our findings can inform future research, Ebola response, and recovery interventions, particularly those targeting community re-integration and mitigation of the fear and stigma associated with survivorship.

## Introduction

Since the identification of the Ebola virus in 1976, Africa has suffered the highest number of outbreaks globally [1]. It is generally estimated that the number of outbreaks are going to increase across Sub-Saharan Africa as habitats for Ebola merge [2]. Over the last two decades, two major outbreaks of Ebola virus disease (EVD) have been recorded: the West Africa outbreak (2013–2016), the largest and deadliest in history, recording 11,310 cases and the Eastern Democratic Republic of Congo (DRC) (2018–2020) outbreak [3,4]. The outbreak in DRC is so far believed to be the second largest and most complicated to manage due to conflict, political instability, poor infrastructure and socio-economic weaknesses causing 2,287 deaths [3,5,6]. Ebola disproportionately impacts people living in rural communities in low income countries causing disruption of economic and social activities [7,8]. The origin of Ebola outbreaks in Africa has tended to be rural and among impoverished communities, including the West Africa outbreak that started in rural Guinea and expanded to affect several West African countries [9–11]. Ebola places a burden on communities and nations through loss of money, economic activities and capital, an increase in unemployment and brain drain, slowdown or closure of health facilities, disruption of internal and external trade, decrease in tourism and travel activity, and an increase in social and cultural barriers [3,11,12].

EVD outbreaks affect many aspects of life at individual, household and community levels [13].While anthropologists emphasize that outbreak response should be guided by fundamental values of decency, dignity, and respect [14], the outbreak in West Africa demonstrated how delays in outbreak recognition, unclear roles and responsibilities among response actors and weak healthcare systems aggravated the overall impact of the outbreak on affected communities [14,15]. Notably, there are other factors that modify the overall response to epidemics and their impact is rooted in a range of historical, political, and social dynamics, including marginalization of certain populations, distrust toward the state, and fears of oppressive or genocidal intervention [16]

The outbreak in West Africa further highlights the outbreak’s impact on the population, health care system and economy of affected countries [17]. Previous studies primarily focused on the disease’s spread, clinical signs, diagnostics, prevention, and more recently, vaccine and drug development [18]. However, this perspective alone cannot fully account for the devastating consequences of the disease on affected individuals and their families [19,20]. Scoping studies have identified the need for comprehensive analysis of the wider social, economic and political impact of the epidemic on the country, communities and survivors [21]. Studies on mental health effects in West Africa have found high prevalence of symptoms related to depression, anxiety and psychological distress due to stigma [13,22].

Stigmatization of survivors affects the ability to establish effective response and recovery strategies following an outbreak [13,23,24]. Disease-related stigma and its sequalae have been documented previously in the context of EVD. The 2000–2001 outbreak in the Gulu, Masindi, and Mbarara districts of Uganda resulted in social rejection, harassment, and discrimination of Ebola survivors [25]. During the 1995 EVD outbreak in Kikwit, Zaire (renamed Democratic Republic of the Congo), EVD survivors often returned to their communities following -recovery to find their homes and possessions destroyed and in some cases, their spouses had abandoned them due to fear and stigma [26]. Community members also refused to buy market goods from EVD survivors affecting their income generating activities and reinforcing marginalization within societal structures [27]. The socio-cultural effects of disease-related stigma can have dramatic effects on housing, criminal activity, meaningful relationships, and individual wellbeing [3,28]. These effects also drive larger, collective factors such as food insecurity, displacement and environmental degradation, which are directly relevant to human interaction with the environment and spillover effects [28].

Uganda has and continues to experience outbreaks of Ebola virus disease (EVD) and the most recent (May 2026, second outbreak due to *Bundibugyo ebolavirus)* is ongoing. Previous outbreaks include the 2022 outbreak in Mubende district that spread to other neighboring districts [29,30]. The country’s first and largest outbreak occurred in the Northern district of Gulu in 2000 [31,32]. This was followed by other outbreaks: 2007 in Bundibugyo district, 2011 in Luwero (single case, one death) and the 2012 outbreak in Kibaale [33]. On 29 November 2007, the Uganda Ministry of Health (MoH) and the World Health Organization (WHO) confirmed the outbreak in Bundibugyo was due to a new stain of Ebola (*Bundibugyo ebolavirus)* [34]. This outbreak concluded with 93 putative, 56 laboratory-confirmed cases and 37 case fatalities [35]. Notably, the 2012 outbreak in Isiro, DRC was retrospectively attributed to the same strain, *Bundibugyo ebolavirus* [35,36]. The majority of cases (36%) in the Bundibugyo outbreak were subsistence farmers and 12% were health workers [37]).

A characterization of short and long term impacts of Ebola outbreaks is a more recent area of focus in research [17,22,38,39]. Ebola survivors often experience long-term physical health problems such as persistent joint pain, headaches, vision impairment, and fatigue, years after infection [37,40,41]. Additionally, they are at higher risk of mental health challenges such as anxiety, panic episodes, sleep disturbances and post-traumatic stress [41].The short-term psychological effects among survivors include the traumatic disease course and the physiological trauma associated with fear of death and proximity to others dying [33,37]. Survivors have also reported feelings of shame or guilt and stigma or blame from others [36,42].These are compounded by a change in cultural practices surrounding many of the traditions that long held communities together [20,36]. At the community level, there is an immediate sense of grief due to loss of community members and shifting family structures due to loss of parents, primary income earners, health workers, community leaders and other social services provision professionals [43]. The United Nations Development Program (2014) predicted an orphan crisis and disruption of longstanding traditional community support in West Africa caused by the Ebola outbreak [8]. However, no parallel studies describe the Ugandan context yet the disease risks particularly in the district of Bundibugyo still remains high as communities’ practices such as eating and use of bats and other wildlife remain high [44,45]

### Purpose

The aim of this study was to understand socio-economic and cultural effects of Ebola among survivors, affected families and communities’ post-outbreak in Bundibugyo district, Uganda. The findings are important in the design of recovery programs targeting Ebola affected individuals and communities. Moreover, our findings add to the emerging body of literature describing the short and long-term impacts of epidemic/pandemic courses in affected communities.

### Conceptual Framework

We used an Eco-health approach integrated with perspectives on disease-related stigma to design the study and interpret reported individual, familial, and community experiences on EVD survivorship in the ten years since the Bundibugyo outbreak. The Eco-health framework is an interdisciplinary approach that recognizes the interdependence of human, animal and ecosystem health and emphasizes that health outcomes are shaped by complex interactions among biological, environmental, social, cultural and economic systems. In studies involving zoonotic diseases, where there are fundamental connections between human, health, and environmental systems, examining factors that potentiate vulnerable points in these relationships offers a greater understanding of the phenomenon as well as potential mechanisms for intervention (Eco-health) [46].

Based on our work and the work of others in the social, cultural, and economic intersections of zoonoses, we framed our analysis through the perspective of Eco-health and stigma. Stigma is a collective, negative social reaction to an individual attribute that is specific to time and place [47]. Depending on contextual factors, enduring disease-related stigma is associated with social rejection, violence, long term inequities in the distribution of income, housing, and health, and significant, negative psychosocial effects of survivorship [39,43,48]. In the context of an outbreak, stigma and resulting discrimination are potentially more damaging than the pathology of the disease itself [43]. In recognition of the transdisciplinary nature of this work, critical realist ontology and epistemology was employed to help derive an explanatory framework of the forces underpinning stigma.

**Interpretive Framework:** Interpretation of themes was informed by a conceptual framework combining an Eco-health approach, the Stigma Framework, and a Critical Realist lens. The Stigma Framework guided analysis of the drivers, manifestations, outcomes, and health effects of stigma, while Eco-health principles situated these experiences within broader socio-ecological relationships, including place, livelihoods, environmental conditions, and human–environment interactions relevant to zoonotic disease contexts. A Critical Realist lens enabled interpretation beyond descriptive accounts to examine the underlying social, structural, relational, and environmental mechanisms through which stigma was produced and sustained in the aftermath of the outbreak.

## Methods

**Fig 1:**
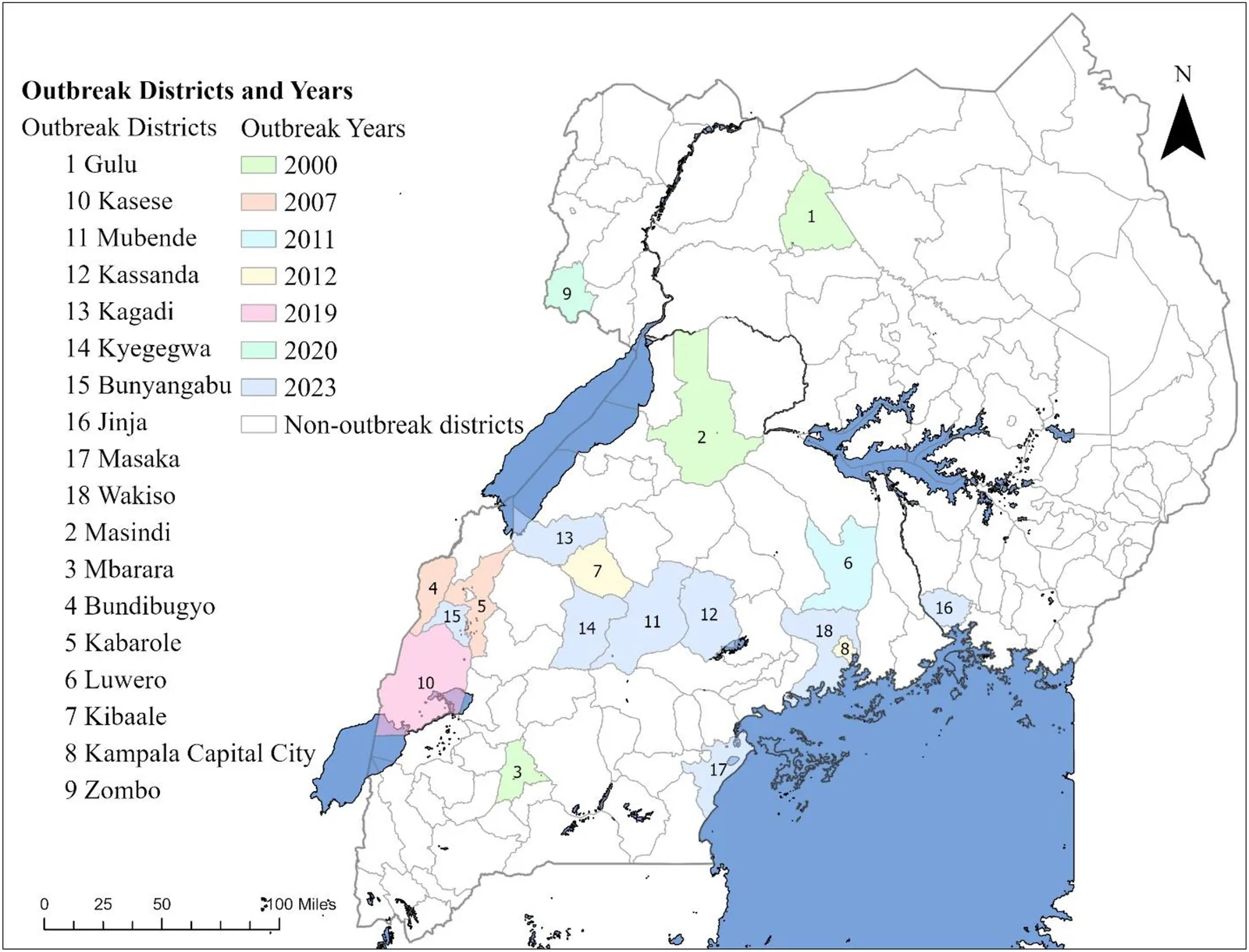
Districts in Uganda with history of Ebola Outbreaks (2000-2023). Cartographic boundaries were obtained from the United States Census Bureau TIGER Geodatabase. Data source: Ministry of Health Uganda reports. Software used is QGIS version 3.44.4-Solothurn.

**Study area:** This study was conducted in Bundibugyo district in western Uganda(Fig 1). It is the only district west of the Rwenzori Mountains covering 850.0 km^2^ and home to a population of 264,778 people [49]. Bundibugyo district borders the DRC in the west and north, Ntoroko District to the northeast, Kibaale district to the east and Kabarole district to the south. The district is relatively isolated from the rest of Uganda and its inhabitants, and their customs are more similar to those of eastern DRC than the rest of Uganda. Moreover, although it is a part of the Nile basin, it is ecologically and culturally part of Central Africa [50]. Data were collected over a period of three months from October to December 2015. The study took place in Bundibugyo town council and Kikyo, about 25 kilometers apart. Kikyo, the epicenter of the outbreak, is characterized geographically by low lying and mountainous areas rising to about 6200–8900 ft above sea level. Its rainfall distribution enables agriculture to take place throughout the year. It is also surrounded by swamps, rivers such as the Humya, and the Semlki Forest, which harbors wildlife including monkeys, chimpanzees, baboons, buffaloes, and other species. [51]. The communities adjacent to the forest practice subsistence agriculture and use the forests to supplement their livelihoods [52]. Some of the forest products include bush meat, herbal medicines, fruits, vegetables, fuel in form of firewood and charcoal, and construction materials such as timber and vines for making ropes [53]. Therefore, the forest is of great social-economic importance to the local communities. Land for agricultural production is steadily shrinking due to an increase in population and the fact that this area is sandwiched between the Semuliki national park and North Rwenzori Forest Reserve.

**Map showing Ebola outbreak areas in Uganda**

**Study design:** We caonducted a focused ethnographic inquiry collecting data through in-depth interviews with survivors, affected families and community members, participant observation, extensive field note documentation and archival data review. The study team consisted of five scholars with veterinary and nursing backgrounds led by an expert in ethnographic research methods.

**Participant sampling:** A total of 19 respondents (Table 1) were included in the study. Participants were categorized as survivors, affected family, and general community members. Survivors were adults who self-reported laboratory confirmed EVD infection, and who were hospitalized and discharged after laboratory confirmed negative status. Affected families were defined as households that had experienced at least one member diagnosed with EVD, regardless of whether the individual survived or died. Community members were defined as residents of the study area who were physically present during the outbreak period and thus able to provide contextual community-level perspectives. The study applied a combination of two non-probability sampling techniques. First, purposive sampling was employed to identify respondents through review of health records at Bundibugyo district hospital and information provided by health workers involved in the outbreak response. This approach ensured the inclusion of individuals with relevant lived experiences of infection, care, loss, and recovery during the outbreak. Subsequently, snowball sampling was used to recruit additional eligible participants. Participants initially identified through purposive sampling, referred the research team to other potential respondents within their social or community networks. This approach was particularly useful in the post-outbreak settings where stigma, relocation, or incomplete records could hinder direct participant identification and recruitment.

**Table 1:** Summary of respondents descriptive statistics (n = 19).

Variable	Category/ measure	n (%)/ value
Location	Bundibugyo—urban	9 (47%)
	Kikyo—rural	10 (53%)
Category	Affected community	6 (32%)
	Survivor	9 (47%)
	Affected family	4 (21%)
Marital status	Married	13 (68%)
	Single	2 (11%)
	Widow	4 (21%)
Sex	Female	5 (26%)
	Male	14 (74%)
Education level	Post-secondary	7 (37%)
	Primary	9 (47%)
	Secondary	3 (16%)
Religious affiliation	Christian (SDA)	17 (90%)
	Christian (Protestant)	1 (5%)
	Moslem	1 (5%)
Age	Mean (SD)	43 (2.4)
	Median (IQR)	43 (32–52)
Occupation	Boda boda rider	2 (11%)
	Businessman	1 (5%)
	District leaders	3 (16%)
	Farmer	6 (32%)
	Health worker	3 (16%)
	Market vendor	1 (5%)
	Social worker	2 (11%)
	VHT	1 (5%)

### Data collection procedures

**In-depth interviews:** Interviews were conducted either in English or local languages. Where necessary, an interpreter was used to facilitate the interview with respondents. The interpreter was a trained qualitative research assistant and a native of the area fluent in English, and the two local languages in Bundibugyo, Rubwisi and Rukonzo. An in-depth interview guide was used to structure and guide the interview process. All interviews were audio-recorded with the consent of participants. In addition, written notes were taken during each interview to supplement the recordings and capture key observations.

**Archival data:** The team reviewed medical records of patients admitted with Ebola at Bundibugyo hospital. These records showed who was affected, the area of residence, ethnicity, religious affiliation, sex, age, dates of admission, treatment received and whether recovered or died. Access to this data was approved by the medical superintendent to facilitate the recruitment of study participants.

**Participant observation:** Data collectors recorded community observation, including activities being carried out, the general home environment, how people interacted with each other, and key issues emerging out of the conversations besides the interviews. All data was collected between November 1 and December 20, 2015.

**Thematic saturation:** After each interview, the research team conducted a debriefing session to review and discuss emerging themes and assess the extent to which new information was being generated. Data saturation was determined through this ongoing analytical process and was considered achieved when three consecutive interviews failed to produce any new themes or insights. The repetition of previously identified patterns suggested that the experiences and effects of the EVD outbreak had been adequately explored, and further data collection was unlikely to enhance the depth or breath of collected data.

**Data management:** Verbatim transcription of the audio recordings was undertaken, and interviews conducted in local languages were translated into English. Investigators reviewed the transcripts with the study interpreter after the translation was complete. Independent verification of all translated transcripts was done by a second study team member who was not involved in the original translation and fluent in one of the local languages and English. Finally, all transcripts were subjected to quality checks against the audio recordings and compared with the handwritten notes by the translator and the investigators.

**Data Analysis:** Thematic qualitative analysis was performed in close collaboration with the research assistant who served as the interpreter during data collection. Two researchers with expertise in qualitative methods independently reviewed a subset of six transcripts and developed preliminary codes using inductive open coding approach. The analysts then compared, discussed and reconciled their independent coding frameworks to develop a shared codebook through consensus. The codebook was then applied to manually analyze the data using Microsoft Word. Analysts constructed coding matrices in which excerpts corresponding to each code were extracted and organized. After coding seven transcripts together revising the codebook iteratively, the two analysts then divided the 12 remaining transcripts equally to complete coding. To maintain analytic consistency, the analysts met after every three transcripts to review and integrate new codes into the coding frame and back code when necessary. Once coding was complete, the codes were then grouped together into categories guided by similarity in content after which the categories were further collapsed into themes. The field notes and archival data were used during data analysis to give context to participants’ narrations, aiding with interpretation. Assessment of intercoder reliability was not undertaken since the study employed reflexive and iterative thematic analysis to make contextualized interpretation of the data rather than to achieve coding uniformity.

Although our research team, comprising veterinarians and nurses who were all external to the study community, brought valuable expertise to understanding the health-related dimensions of EVD, we recognized that our professional backgrounds and outsider status could potentially influence both the conduct of the study and the interpretation of findings. To mitigate this, we worked closely with a research assistant who was a resident of the study area and provided critical contextual insights throughout the research process. Continuous reflexive engagement regarding our positionality enabled regular discussions within the research team and deliberate efforts to minimize the influence of our professional perspectives and socio-cultural backgrounds on data collection, analysis, and interpretation. This reflexive approach enhanced the credibility of the study by ensuring that the findings reflected the perspectives and experiences of the study participants rather than those of the research team.

### Ethical considerations

We received ethical approval to conduct this study from the Joint Clinical Research Center in Uganda and the University of Minnesota Institutional Review Board (IRB Code Number: 1502S62201). Written informed consent was obtained from all study participants. Additionally, Bundibugyo local government, through the office of Chief administrative officer granted the study team permission for community entry. The medical superintendent approved access to medical records and this facilitated the recruitment of study participants.

### Findings

#### Characteristics of study participants.

Table 1 describes the characteristics of the study participants. Their age ranged between 32 and 52 years with the mean and median being 43 years. Majority of the participants were male (74%) while females were about a quarter (26%). All participants reported attaining some level of education ranging from primary school to graduate levels. Whereas men engaged in various income generating activities, all women were engaged with unpaid work in the home. Among male participants, paid employment varied by education level whereby college graduates worked as district technical leaders (16%), health workers (16%) and social workers (11%). Most of those educated below post-secondary level worked as farmers (32%). All participants reported being affiliated to various religious groups with the majority (90%) identifying as Seventh Day Adventists (SDA).

### Description of general life before the Ebola outbreak

Participants described life before the Ebola outbreak as relatively normal and characterized by established social and economic routines. Gender roles largely reflected prevailing community norms, with men primarily responsible for household income generation and women mainly engaged in childcare and domestic responsibilities. Community members participated freely in social gatherings, children attended school regularly, and non-governmental organizations operated within the area, providing humanitarian assistance to address various community needs. Cocoa farming, the principal cash-crop activity in the region, constituted the primary source of livelihood for most households. However, the district faced significant infrastructural challenges, particularly poor road networks. The main road connecting Bundibugyo District to neighboring districts was reported to be in similarly poor condition as the internal district roads, limiting transportation and access to services.

Healthcare services were primarily provided through Bundibugyo District Hospital, which served as the main referral facility for the population and was supported by several lower-level health facilities. Kikyo health center III is where the first Ebola cases in the district were identified.

### Emerging themes

Three major themes emerged during data analysis; i) The Ebola outbreak, ii) The effects of the outbreak, and iii) Life after the outbreak, as described below. Quotes are identified using the interview number and the category of the participant (survivor, affected family or community member). The acronym ‘CHW’ is used for Community Health Workers, and ‘HSP’ is used for health service providers such as nurses, clinical officers, and medical doctors.

### Theme 1: The outbreak

This theme provides a holistic account of the circumstances surrounding the Ebola outbreak, with a particular focus on the interactions between the health care system and the affected community. It has two main subthemes:

**Subtheme 1a: The health care system response** subtheme explores the diagnosis and management of an unusual health event, revealing key challenges in disease surveillance, epidemic detection, and response within a resource-constrained rural context.

**Diagnostic challenges and delayed detection:** Participants’ narratives indicated that both community members and health care workers initially struggled to identify the cause of the illness affecting residents. A recurring observation was the presence of an unfamiliar disease characterized by symptoms resembling those of malaria. Consequently, affected individuals were frequently diagnosed and treated for malaria over a prolonged period before the disease was recognized as Ebola. This diagnostic uncertainty contributed to delays in outbreak detection and the implementation of appropriate response measures.

*“The clinical officer who was very instrumental in diagnosing…had some disease that he called strange of course…A fever, diarrhea and vomiting so anyone would have thought that this was possibly what? Malaria. So he started I think managing, managing, managing but at the end people were not improving.” -* IDI002-HSP

**Low suspicion of EVD:** Despite consultations among healthcare workers, suspicion of EVD remained low. This was partly due to the lack of prior clinical experience with the disease among frontline health workers. More importantly, the previously established typical symptoms of Ebola, bleeding from body orifices, were observed in only a small proportion of cases and occurred during advanced stages of illness. Reports submitted to district and national health authorities did not appear to elicit an immediate response, contributing to delays in outbreak recognition and confirmation. Furthermore, community members’ awareness of Ebola was largely limited to the previous outbreak in Gulu District, which was geographically distant from Bundibugyo, reducing suspicion of local risk.

*“………the way it came was not really typical…people will start thinking of Ebola when you have bleeding from anywhere…the kind of presentation that was here, that would possibly be the latest sign.” -* IDI002-HSP

*“People had no knowledge about Ebola. Of course, people had heard…during the Gulu outbreak but had never had knowledge to know how it spreads, how it looks, of course we used to hear may be on news on how it spreads but being in Gulu which is far away from Bundibugyo.”* **–** IDI003-NGO worker

**Factors complicating early detection:** Some participants suggested that preparations for Uganda’s hosting of the Commonwealth Heads of Government Meeting in November 2007 may have contributed to delays in the detection and official declaration of the outbreak. In addition, the district was concurrently experiencing a typhoid fever outbreak, which may have obscured recognition of an emerging disease with overlapping clinical features. Although cases of what community members described as a “strange disease” had been observed as early as August, the outbreak was not confirmed and declared as Ebola until late October 2007.

*“So, within that period of typhoid fever also Ebola came in so the whole thing was just happening …. I think at one time some of those patients ended in theater thinking that maybe they have gotten perforated gut and they were opened up maybe to find inside there is nothing happening.”* **–** IDI002-HSP

*” It began in August in Kikyo and started getting cases in the last week of September and managed cases in the hospital until 30th October when it was announced as Ebola.”* IDI001- HSP

**Ebola confirmation, fear and panic:** The confirmation that the ‘strange disease’ was Ebola triggered widespread fear and panic throughout the community. Memories of the devastating impact of the earlier Gulu outbreak heightened public anxiety. Healthcare workers experienced levels of fear comparable to those observed among community members, and some professionals who had been working in the district chose to leave. This resulted in disruptions to healthcare service delivery and weakened the social and economic systems that were essential for supporting outbreak response activities.

*“Most of the staff in the ward and even patients fled due to fear…… That experience was really terrible for me and my colleague. Even my family members often pestered me to leave unit/hospital but I told them no.” –* IDI001- HSP

*“The bank was the only way to get…support in terms of funds to run these activities. But you are going to the bank, the bank manager, everybody in the bank they are scared.”* – IDI003-NGO worker

**Subtheme 1b: The affected communities’ response:** This subtheme describes community perceptions and responses during the emergence of an unidentified disease outbreak, in the context of limited diagnostic capacity and uncertainty within the healthcare system.

**Emergence of alternative explanatory narratives and associated social tensions:** The absence of a definitive diagnosis and clear public health guidance led community members to develop various explanations for the disease outbreak. Some participants attributed the illness to witchcraft, particularly because multiple cases appeared to occur within the same families. Others interpreted the outbreak as the result of ritual sacrifice or deliberate harm by certain community members. These interpretations contributed to social tensions and, in some instances, resulted in acts of violence directed toward individuals suspected of involvement.

*“People were thinking that maybe there are some charms and there was a story somewhere that someone there is a witch doctor so people were thinking that maybe because he was getting some people from Congo coming for those herbal medicines.”-* IDI008- Survivor &HSP

*“One home in Butholya six people died at once. There was a young rich man in that village, they thought he is the one sacrificing them, even on the burial people had to beat him. It was after the death of the 5th person and people thought that maybe he is getting……he wanted to get money using “mayembe” (local charms), up to now that man shifted to Kasese and said he will never come back, he hates the whole clan and the family.” -* IDI018- HSP

**Reinforcement of pre-existing inter-ethnic tensions:** Although tensions between the two dominant ethnic groups in the study area predated the Ebola outbreak, uncertainty surrounding the unidentified disease appeared to exacerbate existing divisions. Participants reported narratives suggesting that members of the Bamba community, who reside at the foothills of the mountain, had contaminated water sources used by the Bakonzo community, among whom most early cases were reported. These suspicions persisted until the disease was laboratory-confirmed as Ebola, after which such claims largely subsided.

*“I remember at first there was general complaint that there is a strange disease and it is killing people. You know here we having majorly like two or three tribes; the Bakonzo, the Bamba, the Babwisi and this strange disease was in the area dominated by the Bakonzo. So, I remember they came to our office, the CAO’s office complaining that, the Bakonzo have been poisoned, you know.”* – IDI014-Government official

**Acceptance of control measures and compliance:** Following the official declaration of the Ebola outbreak, community responses shifted toward greater acceptance of the disease and compliance with recommended public health measures. These responses seemed to be strongly influenced by knowledge of experiences from previous Ebola outbreaks in other parts of the country. Fear of infection coupled with heightened risk perception pushed community members to become increasingly vigilant and cooperative with outbreak response efforts, including reporting individuals with suspected symptoms to health workers.

*“We’ve heard of Ebola in Sudan; we had heard of Ebola in Congo so the moment people heard this is Ebola there was no one like to contest it. People knew this was Ebola the only problem now was everyone was starting to fear for him/herself”* **–** IDI002-HSP

**Use of unconventional preventive remedies:** Besides complying with official public health measures, community members adopted various self-protective practices aimed at reducing their risk of infection. Some participants reported excessive use of sodium hypochlorite (JIK) for disinfection purposes, resulting in adverse health effects such as skin burns and abdominal discomfort among individuals who ingested it. Participants also described increased consumption of waragi, a locally produced alcoholic beverage, based on the belief that it possessed protective or cleansing properties. This perception was reportedly influenced by observations that some individuals who consumed the beverage remained unaffected by the disease or survived it.

*“There were three beliefs (about the relationship between waragi and Ebola); if someone was a drunkard, he could not die of Ebola, because it’s only those who had never tasted alcohol that died and only those who were known drunkards survived. Waragi being a spirit helps to clear the stomach and Ebola was associated with taking dirty things. and lastly that drinking makes people not to move, so you stay in the same place reducing the risk of getting outside.”* **–** IDI019-Affected family

### Theme 2: The effects of the outbreak

The Ebola outbreak and the measures implemented to contain it disrupted routine social interactions and community life. Fear of infection, coupled with public health restrictions, influenced interpersonal relationships, social participation, and access to support networks. This theme explores the immediate social consequences of the outbreak at the individual, household, and community levels.

**Subtheme 2a: Stigmatization and discrimination by society:** Containment measures introduced during the outbreak had significant implications for the social lives of community members. Restrictions on social gatherings, cultural events, and ceremonies limited social interaction and contributed to feelings of isolation among affected individuals and households. Participants reported that individuals who had been in contact with Ebola patients, as well as their family members, often experienced social exclusion, avoidance, and rejection by other community members due to fears of disease transmission.

*“Completely no, you couldn’t do a wedding. Those are the things you could not talk about. What was here was basically fighting the disease and looking for what to eat. These other social issues, we would not talk about them”–* IDI014-Government official

*“… One day one of the patients died and everyone was fearing to carry the body. For me I decided I would do it after all the body was already in the coffin. My other friend also did the same on a different day and then people started fearing us that those ones carry Ebola, saying that’s the one who carried Ebola he is going to affect us, they would run away whenever they would see us at a distance. We used to go to restaurants and they would refuse to give us food because we carried bola patients and dead bodies.”* **–** IDI015-Boda boda rider

**Disruption of the social support systems:** Uncertainty regarding the infectiousness of Ebola survivors contributed to persistent stigma directed toward survivors and their families. Participants described widespread fears that survivors remained capable of transmitting the disease as a factor that hindered the provision of care and support to individuals who were in need.

*“There was a day I came to town to cut my hair and the people scampered away saying I am an Ebola patient. People used to run away from us.”* – IDI006-Survivor

*“It was the father who used to take care of the baby. I did not have anyone else to take care of me. He used to carry the baby while putting on gloves. He would take the baby and bring him at a time of breast feeding. My husband had to take that risk as a parent; he couldn’t throw me away* **–** IDI017-Survivor

**Subtheme 2b: Disruptions to cultural practices and rituals:** The Ebola outbreak and associated containment measures resulted in temporary modifications to several cultural practices within the community.

**Disruption of burial practices:** Participants reported substantial changes to customary burial and funeral practices during the outbreak. Most families were permitted to bury deceased relatives on their own land under the supervision of trained burial teams. Exceptions included certain categories of individuals, such as healthcare workers and religious leaders, who were buried at designated sites associated with health facilities and places of worship, respectively. While these measures facilitated safe and dignified burials, participants noted that restrictions on traditional funeral rites and mourning practices left many family members with unresolved grief and emotional distress.

*“He (my husband) was not buried in the normal way, they did not wash him, they just brought him from the hospital after they had wrapped and put him in the coffin direct into the grave, they did not perform any rituals. Too bad but there was nothing to do, it was touching, because I could not burry him in the normal way.”* **-** IDI005-Survivor

To minimize the risk of disease transmission, communities residing in lowland areas also provided land for the burial of individuals from mountainous regions. This arrangement reduced the need to transport bodies over long distances in settings where motorized transportation was limited, supporting outbreak control efforts.

*“People were scared. Others would be buried on the way if a vehicle can’t reach your home. They would request any relative who has land where a vehicle can reach to bury there. People would feel bad but there was nothing to do, because we feared the disease, no one could be blamed.”* – IDI016-Survivor

**Temporary changes in hunting practices:** Participants also described temporary changes in hunting practices during the outbreak. Hunting activities declined substantially following widespread public health messaging linking Ebola transmission to contact with wild animals, including non-human primates such as monkeys and gorillas. These behavioral changes were largely short-lived, however, with participants reporting a return to customary hunting practices after the outbreak was officially declared over.

*“People forget easily, our communities forget easily, because if somebody must have eaten a monkey, I should say at this time you cannot stop him if he is still living, if he is a hunter, he is still hunting…Even the animals in the bush were like, why are we not being hunted this time because there was negativity because of the constant information people had to change their way of living.”* **–** IDI003-NGO worker

**Subtheme 2c: Family level consequences:** While the outbreak had widespread community-level effects, its most immediate and profound impacts were experienced within households. Participants described significant emotional distress, grief, and disruptions to family life, particularly among families that experienced multiple Ebola-related deaths. The loss of several household members compounded the burden of bereavement and altered family dynamics, caregiving arrangements, and social support systems.

*“Very many people died. You know where there is death, it is a disaster…some families where both a father and a mother died, some families a father died, some families a mother died, so as you know where there is death there automatically must be some challenges which are negative to the lives and of course being that the end of it all who are the victims of such a situation? There are the children. Most children were left orphan and now they had no people to look after them.”* **–** IDI003-NGO worker

**Disruptions to family relationships and social connectedness:** The Ebola outbreak affected family relationships and social networks in multiple ways. Frontline healthcare workers involved in case management frequently experienced prolonged separation from their families due to infection prevention measures and concerns about disease transmission. Participants also reported the loss of friends and significant others during the outbreak, contributing to emotional distress and social disruption.

*“I was cut off from the family because now as I recovered the third week I joined the isolation. I couldn’t even go home I was just at the isolation site. Then after that aha 21 days they monitored me at least that one I went home”* **–** IDI008-Survivor&HSP

Within households, measures aimed at reducing the risk of infection altered customary patterns of interaction. For example, some men in polygamous unions reported limiting contact to a single spouse as a precautionary measure. Participants also described restrictions on sexual relations between partners, particularly where one partner was an Ebola survivor, reflecting concerns about potential transmission and adherence to public health guidance. Fears surrounding exposure to the virus created tension within marital and household relationships. In some cases, these tensions resulted in temporary or permanent separation between spouses.

*They said that I had to spend three months without sleeping with my wife. I was put in a separate room. So from there I was give some condoms so that if I feel that the situation was unbearable, I should use a condom.* **–** IDI006-Survivor

*“My own wife run away. I decided to leave her because you go when am not sick, what will happen if I became sick? It means you can’t take care of me, we had to separate. I have never even got another one I decided to leave women, that woman made me leave women.”* **–** IDI015-Boda boda rider

**Subtheme 2d: Economic and livelihood effects:** The Ebola outbreak disrupted local economic activities and livelihoods, affecting the production, distribution, sale, and consumption of goods and services. Participants described reduced economic productivity, restrictions on trade and movement, and loss of income at both community and household levels. The extent of these impacts varied according to occupation, gender roles, household responsibilities, and direct exposure to the outbreak.

**Loss of income from cocoa cash crop:** Bundibugyo District is a major cocoa-producing area, and many households rely on cocoa farming as their primary source of income. The outbreak coincided with the cocoa harvesting season disrupting income generation and trade given movement restrictions and fears of disease transmission. As the district became socially and economically isolated from neighboring areas, trade in agricultural produce and general merchandise declined substantially, further limiting producers’ market access and reducing household incomes.

*It came at a time when you know this place has a major cash crop called cocoa and there is a season actually that is when it begins from around September, October, November, December, January that’s when the season is peaking.* – IDI002-HSP

*“The economy… there was no business, everywhere business came to a standstill of course who would buy? People were just at home, everybody was scared. You know people would even think that by merely moving, one would probably contract Ebola, but also once you move a head of household would tell you, whoever moves should not come back here, just stay there, so there was no movement, no business.”* – IDI003-NGO worker

**Disruption to local businesses:** Participants reported that individuals engaged in business and transport-related occupations were particularly affected due to reduced mobility, market disruptions, and declining customer demand. Men, who were often regarded as primary income earners within households, described difficulties in meeting household needs during and after the outbreak. Ebola survivors involved in income-generating activities also reported challenges in rebuilding their livelihoods and providing for their families following recovery.

*“My customers first feared me. It took me about six months and my business was really slow. People used to say they fear me, I still have Ebola….It (business) was closed because if my wife had opened, they would not have given her money, she was also feared & all my people at that time. They feared my family as well…Almost if we could add the months that I spent in the hospital without working, plus the time I spent home, it’s almost three years. The three years were difficult.”* **–** IDI006-Survivor

### Theme 3: Life after the outbreak

This theme presents how the affected community has readjusted to life after the disruptions associated with the outbreak including long-term effects of the outbreak.

**Subtheme 3a: Deepening tribal conflict:** The two main communities, the Bakonzo and Bamba, still traded blame long after the outbreak.

*“Okay, as I talk even now there still those clashes on small scale between the Bakonzo and the Bamba. Other people claim the district is theirs, others claim to be from Kasese so there is no real rich understanding of the point”. -* IDI008- Survivor &HSP

**Insults linked to bush meat consumption:** The Bamba ethnic group still believed that the rival Bakonzo tribe who live in the mountains were responsible for the Ebola outbreak and its associated devastation.

*“The ethnic group which was more affected were Bakonzo…After the ministry of health declared some causes of Ebola disease that it was caused by eating some monkeys and …because of neighboring the Rwenzori mountains where the monkeys stay that it was due to that, they are hunters that’s why they were affected, such a kind of statements are painful* – IDI012-CHW

**Subtheme 3b: The road to recovery:** The recovery process varied across individuals and families depending on whether the person was a survivor, affected family or a community member.

**Psychosocial support:** To support recovery, immediate psychosocial and material assistance was provided to survivors and affected families. Community engagement activities, including open discussions, were conducted to promote understanding of the importance of supporting survivors and to address misconceptions regarding the potential transmission of the virus by recovered individuals. As community members gained a better understanding of these issues, acceptance of survivors increased, facilitating their social reintegration and contributing to the recovery process.

*“People started knowing that although it is a deadly disease, we can manage. Each one had different feelings. Especially those who lost relatives were grieved for a long time and wondered if they would be compensated. The hardest thing was the psychological trauma… they were very sad and lived with no hope for a long time.”* – IDI001-HSP

*“In the first days and weeks, some of the families feared their fellows after discharge and it took time for them to accept or be in contact with them. After some time, they realized that these people were not contagious. In fact, if you go to the affected homes, you wouldn’t know them not unless they tell you.”* – IDI001-HSP

However, despite the support provided, some affected families perceived the assistance as insufficient. Widows who lost their husbands, often the primary household breadwinners, were particularly vulnerable, with some still experiencing psychological distress and trauma years after the outbreak. In addition, older adults who lost their children faced the ongoing challenge of raising their grandchildren in their old age. Several survivors and affected families also expressed the view that they should have received compensation for the loss of household belongings and other material assets.

*“The widows still manifest some kind of sadness. Especially one widow has never come back to the normal life. When you look at her she still has some sadness. She is somebody who was depending on her husband and she explains that she has lost that relationship and her way of life has not come back to normal.”.* – IDI001-HSP

*“We lost him (husband) … we are suffering, I am suffering, I am taking care of myself and the kids and I don’t have any support.”* **–** IDI009-Affected family

**Chronic health conditions:** Survivors experienced a recovery trajectory characterized by both psychological and physical sequelae, further complicated by the trauma they faced while in the isolation ward. Commonly reported symptoms include generalized body weakness, headache, hypertension, pneumonia, diabetes, and impaired vision.

*“I just knew that I am already dead. You can imagine another patient dying when you are just seeing and you are suffering from the same disease! We were not allowed to get out of the ward, they would keep it closed all the time. I couldn’t think that I can survive.”* IDI016-Survivor

*“The way I see since I got hypertension & diabetes because of too much thinking…I used to think that am dead, am no longer among the live ones. That’s how I think Ebola changed my life. Otherwise, am okay.”* – IDI006-Survivor

*“I have some eye problems, in the morning hours I don’t read very well, that was a after the disease, so I use some glasses to read properly. Then periodically I normally get, we call it lobe pneumonia, it’s normally one side so that one comes at least every month at the beginning of the month I experience that thing.”* – IDI008-Survivor&HSP

**Improved emergency preparedness and health-seeking behavior:** The outbreak had some positive effects on the medical community’s preparedness capacity, illustrated by the improved ability of healthcare workers to adhere to infection control measures. Similarly, community members made healthier decisions and adopted a more proactive approach to seeking medical attention for unknown or suspicious health conditions they encountered.

*“Before Ebola, some health workers took the use of gloves for granted but now they know the importance of hand washing and use of protective equipment. We have some protective gadgets in the store awaiting any events. Before Ebola we had traditional healers, even now they are there. Some of the patients were rushed to the traditional healers. Whereas they are there, when the community suspects that the disease is unknown, they bring the patient to the hospital.”-* IDI001-HSP

*“When I fell sick of Ebola, I realized that anytime I could die. I realized that now I need to plan for my future because I reached the extent of death. I used to take alcohol before Ebola but after I returned from hospital, after my dad died, I sat down and asked myself, nobody gave me medicine to leave alcohol. I asked myself a question, “I have been taking this alcohol for long, what Have I benefited?”. I replied myself and decided to quit alcohol.”* – IDI004-Survivor

**Subtheme 3c: The unanswered questions:** Most of the participants had vivid memories of the situation during the outbreak and had questions regarding the viral disease emphasizing the need for more research.

**Are survivors immune to Ebola?** Survivors expressed desire to know whether they had acquired natural immunity and were safe to participate in helping others in case of a future outbreak.

*“Like me I would have loved that when you do such research to find out that among us who suffered from Ebola, can’t we catch it again? I want you to do research in that area because another disaster might happen and we go there saying we survived let’s help others & end up getting the disease again. Do research on that if a person who suffered Ebola cannot catch the disease again. Do research on that.”* **–** IDI006-Survivor

**Where and how did Ebola start?** Community members expressed persistent uncertainty of where the disease first emerged from. Given the outbreak’s unprecedented nature in this area, rumors, lay observations and partial public health messaging formed the bedrock of community decision making. Two origin narratives were especially prominent; one linked the outbreak to a hunting a hunting community near the forest, after multiple members of a family living in close proximity to the forest died. A competing theory attributed the origin of the outbreak to contact with a patient from the DRC who had visited a traditional healer. This lingering confusion appears to have contributed to persistent fear that Ebola could return.

*“It has been difficult to establish the origin but it started in Kasitu Sub county currently Ngamba Sub county. There are many versions about the origin. Some people say there is a family that ate a monkey and another story in Ngamba then was that a family where the family leader is a traditional healer had a patient who came from Congo and was managed and died.” -* IDI001-HSP

*“Some people ate bush meat but these are all speculations…They used to tell us during their campaigns here that Ebola is not airborne and I think that is what they say. Isn’t it? That you will not get it by someone sneezing on you…you get by contact with someone body fluids…. they just think...”* – IDI002-HSP

**How come children were not affected?** No children were reported to have contracted or died from EVD, including the child of a mother who continued breastfeeding while ill with Ebola and after recovery. Although this finding is based on a single reported case, it highlights the need for further research into the dynamics of breastfeeding and the risk of Ebola virus transmission from mother to child.

*“They used to bring the baby in the evening, and we sleep together, and they come for him very early in the morning and baby didn’t get the disease. I would breast feed him very early in the morning then they take him the whole day. They would give him porridge and milk during daytime. He was like 3 months old that’s why I had to breast feed him.”* – IDI017-Survivor

**Memories, emotions, and perceived risk of future outbreaks:** Several years after the outbreak, most individuals had largely resumed their normal daily lives, with the notable exception of bereaved family members and survivors who continue to experience long-term physical and psychological sequelae. Ebola is now widely known within the community, with a level of familiarity comparable to that of malaria. When participants were asked about their reactions to hearing the term “Ebola,” responses ranged from indifference or absence of fear to strong emotional reactions characterized by fear, grief, and memories of substantial loss of life during the outbreak. Participants also expressed diverse views regarding the possibility of future outbreaks. Many described the Ebola outbreak as the most traumatic event they had experienced and indicated a strong desire to avoid reliving a similar experience.

*“I feel shocked, it’s like death, that people will die again the way it happened, it was not an easy situation. It is not easy disease” and to others “A dragon monster again because of all the people who have come for research, they have told us that they have not come up with medicine to treat Ebola. So, it is a scaring word once you hear Ebola then that’s a scaring word because it’s something that cannot be treated.”* –IDI013-Administrator & affected family

*“This time if they say that Ebola has come, I will leave boda boda business, eeeh! The truth is that the disease is dangerous*.” **–** IDI015-Boda boda rider

## Discussion

Over ten years down the line, communities in Bundibugyo still live with the effects of the 2007 Ebola outbreak that occurred in their area. This study examined the socio-cultural and economic effects of an Ebola outbreak on survivors, affected families and communities in Bundibugyo and at the time of the study, the outbreak in West Africa was on-going. Three major themes emerged during data analysis; i) The Ebola outbreak, ii) The effects of the outbreak iii) Life after the outbreak.

Host–parasite interactions are a key driver of infectious disease emergence and transmission. For human hosts these interactions transcend biological factors to include the social cultural environment. Ebola is a disruptive disease that occurs at the intersection of culture and public health [54]. It’s therefore important that we include social science approaches to explain the cultural, social and the economic impacts of such outbreaks. Studies have shown that there are many social, cultural and economic changes that occur during and after the outbreak placing a significant burden on the lives of affected people [8,48] Although this outbreak was caused by a mild strain [55], the community had never witnessed people dying in large numbers before. The community response in this outbreak was driven by fear, shaping the response mechanisms before and after the confirmation of the outbreak. Fear, panic and anxiety in the community was attributed to the overall crisis communication management that was used, initially inadequate due to the strange nature of disease as studies have indicated [56]. This affected the social cultural fabric and by extension the disease dynamics particularly during the outbreak. There is fear that communities in Bundibugyo continue to live with, the fear that the disease could strike again, pushing them to continually seek for a proper answer to what caused the disease. This experience is similar to what has been described in the Kibaale Ebola outbreak, where constant fear and lingering pain of the experience has been described [57]. Therefore, this calls for the long-term investment in research and engagement with communities to try and understand the origin of the disease, factors that could potentially lead to outbreaks and how best to support long term community recovery. However, just like in many other Ebola outbreaks in West Africa, DRC including the most recent outbreak in Uganda, the origin remains unknown [30,58].

Several studies have linked hunting and bushmeat consumption to Ebola outbreaks [59,60]. Human contact with wildlife species, both direct and indirect, is undoubtedly an important factor in the transmission and emergence of new human pathogens from wildlife [54]. This contact is further exacerbated by the conflict with the land tenure system [61,62]. However, communities in Bundibugyo continue to hunt, eat, and trade in bushmeat and have highlighted several health and economic benefits that accrue from this practice. This finding is similar to findings in Western Uganda [63]. This is further highlighted by rejection of messaging that unilaterally stressed the health risk posed by wild meat that contradicted the experiences of target publics in West Africa Ebola outbreak, who consume wild meat without incident [59]. The communities in Bundibugyo are asking for explanations as to why hunting and bushmeat consumption are risk factors for spillover now and not before, since these are practices that have been going on for generations. It’s important to note that risk perception among such communities remains low [64], this calls for concerted efforts among stakeholders to involve communities in the management of zoonotic diseases for early detection of such outbreaks [65,66].

Outbreak detection can also be enhanced by availability of in- country diagnostic facilities, a key response function of the healthcare system. In the Bundibugyo outbreak, Ebola samples were tested in the USA and delays in laboratory confirmation meant that the strange disease was initially treated as malaria because of similar clinical presentation until a definitive diagnosis was made a [67,68]. Communities also tried to seek for alternative treatment for the strange disease using witchcraft and acts of sacrifice. Tribal conflicts also played a role as Bakonzo accused the Bamba of poisoning their water source. However, these (witchcraft, acts of sacrifice and tribal accusation) resolved when the Ebola outbreak was confirmed and communities united to confront a common enemy, Ebola. This is contrary to what has been reported in the West Africa Ebola outbreak, where there was conflict between health workers and the community due to violation of cultural beliefs that are usually contradictory to prevention measures, demonstrating the value of continuous community engagement in the management of such outbreaks [69]. Lessons from COVID-19 further indicate that, heavy-handed bans risk numerous unintended consequences and tend to fail if they lack legitimacy or clash with people’s values [70]. Analytic studies have therefore made wide recommendation to countries to assess existing community engagement structures and use community engagement approaches to support contextually specific, acceptable and appropriate COVID-19 prevention and control measures [71]. Success stories however exist like in Vietnam where citizen trust in government, citizen and social organizations’ engagement reduced the impact of COVID-19 [72].

During the outbreak, communities’ response mechanisms also involved consumption of alcohol and jik to prevent the disease. The consumption of alcohol for example was supported by community observation that some survivors were alcohol users and most of those that died, were non-alcohol users because of their religious belief. Communities believed that alcohol was a cleanser for the “Ebola germ” and offered protection to consumers. There were reports of people sustaining serious burns from jik and although many participants believed the consumption of alcohol was helpful in preventing the disease, it remains a speculation. This highlights ongoing community learning process with detrimental or beneficial effects, that is rarely documented in such outbreaks. It also highlights opportunity for partnership between local communities and use of their local observations that could be a source of insight into protective or beneficial strategies that can be taken advantage of in the battle against such emerging infectious diseases [44]. For example, in most rural communities’ consumption of alcohol is accompanied by copious consumption of meat as well [73]. Perhaps the differential health outcomes between the alcohol and non-alcohol users who are exposed to the Ebola virus could be tied to this varied access to protein between the two groups.

Overall, the effects on survivors, affected families and community varied from side effects, a sense of loss, physical and psychosocial effects among survivors and deepening tribal conflict persisting for years in some cases as already found [37]. Many survivors reported some positive outcomes such as improvement in personal hygiene and protection practices, self-awareness and health seeking behavior. However, the negative impacts were more. They include self-isolation, stigmatization. shame and embarrassment, and loss of close relatives similar to what has been reported in West Africa and DRC [39]. Indeed, stigma is a pervasive challenge in public health, particularly during infectious disease outbreaks such as EVD and some of the long term sequelae associated with the disease [13]. In this community it manifested as a complex interplay of structural, cultural, and individual factors that impact health outcomes and community resilience. Using a Critical Realist lens, the Stigma Framework, and the Eco-Health approach, and based on the findings from this study we distilled the drivers, manifestations, outcomes, and health impacts of stigma at play in this community. This narrative synthesizes these perspectives to provide a comprehensive understanding of stigma’s role in shaping responses to EVD within this community. For instance, fear of infection drove heightened anxiety about contracting Ebola leading to avoidance behaviors that manifested as social ostracism. While concerns about livelihood loss and societal rejection further exacerbated stigma, which were in turn fueled by a fear of infection. Unsurprisingly, this fear of infection festered and thrived within a context of misinformation both about the pathogen/patients and survivors. Particularly given some of the cultural beliefs about at-risk individuals or communities, leading to prejudice, physical rejection, discrimination of survivors and ethnic tensions. All this occurred within a context of mixed or unclear messaging and/or delayed or inadequate government interventions further amplifying fear and uncertainty. These observable stigma practices are identified by the critical lens as emerging from underlying mechanisms that would include systemic inequities in resource distribution and health care access, or land use strategies, driven by livelihood pressures, that fuel zoonotic spillover events [74].

Most significant is the impact on widows who lost their husbands. The young widows felt a great sense of loss and burden to support their children to be able to go through school. On the other hand, the older widows were haunted by the fact that their spouses did not get a decent burial and many reported withdrawing from community related activities (Self-isolation). Disease response measures can interrupt education in the short and long term. During the 2014 Ebola epidemic in West Africa and the 2018 Ebola outbreak in DRC, schools were closed, or parents were reluctant to send their children to school, due to fear of contagion. Additionally, widowed women after the loss of their spouse, struggled to take care of their children’s school needs, widows were also barred from ‘accessing their deceased husband’s land because of discriminatory inheritance laws [75], further revealing the pernicious long term effect of the disease. Effects that transcend the epidemiological triad and have far-reaching negative impacts on the social fabric of these communities.

### Study limitations

A key strength of this study is that data were collected several years after the Ebola outbreak, allowing for the exploration of both immediate and enduring consequences of the epidemic. This extended post-outbreak timeframe provided a unique opportunity to capture long-term social, economic, and health-related impacts that may not have been apparent had data been collected shortly after the outbreak. As such, the study contributes to the growing body of evidence highlighting the persistent effects of epidemics on affected populations, effects that are often overlooked once disease transmission has been controlled and public health attention shifts elsewhere. Furthermore, the findings underscore the value of meaningful community engagement throughout the research process with the recognition that people with lived experience can inform future research agenda as demonstrated by the pertinent questions the respondents raised.

Despite these strengths, several limitations should be acknowledged. First, because data were collected many years after the outbreak, the possibility of recall bias cannot be entirely excluded. Participants may have experienced difficulty accurately recalling certain events, perceptions, or experiences related to the outbreak. However, several measures were taken to mitigate this risk, including the triangulation of findings across multiple data sources and participant groups. In addition, the Ebola outbreak represented a highly significant and disruptive life event for affected individuals and communities, making key experiences and memories more likely to be retained over time. While recall bias remains a consideration, we believe its influence on the overall findings was minimized through these methodological approaches. Finally, as with many qualitative and context-specific studies, the findings should be interpreted within the social and geographic context in which the research was conducted, although they may offer important insights for understanding the long-term consequences of epidemics in similar settings.

## Conclusions

Nearly a decade after the 2007 Bundibugyo Ebola virus disease (EVD) outbreak, survivors, affected families, and communities continue to experience significant social, cultural and economic consequences. While some positive changes like improved hygiene and health-seeking behaviors were reported, persistent stigma, fear, psychosocial distress, social isolation and livelihood disruption remained the dominant long-term impacts. These findings demonstrate that recovery from EVD extends beyond the end of the outbreak. This study therefore highlights the importance of integrating social science in responding to both the short- and long-term needs of communities hit by these emerging infectious diseases. Long term investment in community engagement, stigma reduction, psychosocial support, and livelihood support are essential to strengthen recovery and improve preparedness to future outbreaks.

## Supporting information

S1 File Inclusivity-in-global-research-questionnaire. (PDF)
