# Supplementary material for: Fear, stigma and Uncertainty: The short and long-term effects of Ebola on survivors, affected families, and community in Bundibugyo, Western Uganda
Source: PLoS One. 2026 Sep 8;21(9):e0355778. doi: 10.1371/journal.pone.0355778 (PMC13552781; doi:10.1371/journal.pone.0355778)
Supplement: Supplementary file 1 [file pone.0355778.s001.pdf]

Inclusivity in global research

PLOS' policy on inclusivity in global research aims to improve transparency in the reporting of research performed outside of researchers' own country or community and ensures that PLOS publications reporting global research adhere to high standards for research ethics and authorship. Authors of relevant research articles may be asked to complete the questionnaire below, which outlines ethical, cultural, and scientific considerations specific to inclusivity in global research. This questionnaire may be requested when researchers have travelled to a different country to conduct research, if research uses samples collected in another country, research with Indigenous populations or their lands, or if research is on cultural artefacts. Researchers travelling to another country solely to use laboratory equipment will not normally be required to complete the questionnaire. However, the questionnaire can be requested at the journal's discretion for any submission – if you have been requested to complete this questionnaire by the PLOS journal you submitted to, please do so.

Please complete the questionnaire below and include this as a Supporting Information file with your manuscript. Note that if your paper is accepted for publication, this checklist will be published with your article in the supporting information files. Please ensure that you reference the checklist in the main body of your manuscript. We suggest adding a subsection 'Inclusivity in global research' to your Methods section and adding the following sentence: "Additional information regarding the ethical, cultural, and scientific considerations specific to inclusivity in global research is included in the Supporting Information (SX Checklist)"

The questions have been designed to be applicable to a wide range of study types, and there are subsections for both human subjects research and non-human subjects research. If any of the questions are not relevant to your research please mark them as "N/A" as appropriate.

Ethical considerations, permits and authorship

This section is applicable to all research types.

Provide details as to who granted permissions and/or consent for the study to take place in the Methods section of your manuscript. This should include the names of **all** ethics boards, governmental organizations, community leaders or other bodies that provided approval for the study. If individuals provided approval refer to these people by their role or title but do not list their name(s).

Reported on page number 7: We received ethical approval to conduct this study from the Joint Clinical Research Center in Uganda and the University of Minnesota Institutional Review Board (IRB Code Number: 1502S62201). Written informed consent was obtained from all study participants. Additionally, Bundibugyo local government, through the office of Chief administrative officer granted the study team permission for community entry. The medical superintendent approved access to medical records and this facilitated the recruitment of study participants.

If there were any deviations from the study protocol after approval was obtained please provide details of these changes in the Methods section of your manuscript.

Reported on page number: No deviations from the study protocol

Did this study involve local collaborators that are residents of the country where the research was conducted or members of the community studied? If you do not have any authors from said communities, please provide an explanation for this below.

Yes. Two of the authors (Shamilah and Jacinta) were residents in the One Health USAID-funded program coordinated through the One Health Central and Eastern Africa (OHCEA) network. In addition, local leaders and community members facilitated community entry and participant recruitment. The study also employed a trained qualitative research assistant who was a native of the study area and fluent in English, Lubwisi, and Lukonzo.

Everyone listed as an author should meet PLOS' criteria for authorship and all individuals who meet these criteria should be included in the author byline, rather than the acknowledgements. For further information please see the journal's Authorship Policy.

Human subjects research (e.g. health research, medical research, cross-cultural psychology)

Did you obtain written informed consent from a representative of the local community or region before the research took place? How did you establish who speaks for the community? Details of written informed consent obtained from study participants should be reported separately in the Methods section of your manuscript.

Prior to conducting research at the community level, written permission was obtained from the relevant local authorities, in this case the Bundibugyo District Chief Administrative Officer (CAO). The approval process involved submitting an official introduction letter from OHCEA, which provided a detailed explanation of the study objectives and planned activities, together with Institutional Review Board (IRB) approval letters from the University of Minnesota and the Uganda Joint Clinical Research Centre (JCRC).

The submitted documents were reviewed and officially stamped by the CAO's office. Upon approval, the CAO signed the authorization documents. One set of the signed and stamped documents was returned to the research team as evidence of local authorization, while another set was retained by the CAO's office for official records. The authorized documents were subsequently presented to local leaders before interviews were conducted.

How did members of the local community provide input on the aims of the research investigation, its methodology, and its anticipated outcome(s)?

Community members did not directly contribute to the formulation of the study aims, methodology, or anticipated outcomes. However, during interactions with the research team, community members expressed a strong interest in learning about the study findings and their implications for future outbreak preparedness and response.

When engaging with the local community, how did you ensure that the informed consent documents and other materials could be understood by local stakeholders?

A trained qualitative research assistant, who was a native of the area and fluent in English, Lubwisi, and Lukonzo, served as an interpreterry. The interpreter assisted not only during interviews but also in explaining the study objectives, informed consent procedures, participant rights, and any study-related information to ensure accurate understanding.

Before obtaining consent, participants were given the opportunity to ask questions and seek clarification regarding any aspect of the study. The research team confirmed participants' understanding before proceeding with enrollment and data collection. This approach helped ensure that informed consent was genuinely informed and was both culturally and linguistically appropriate for all participants, regardless of their level of formal education or English proficiency.

Will the findings of the research be made available in an understandable format to stakeholders in the community where the study was conducted (e.g. via a presentation, summary report, copies of publications, etc.)? Please provide details of how this will be achieved.

Yes. A copy of the publication will be shared with the Bundibugyo District technical team through the Bundibugyo District One Health Committee and the District Outbreak Response Team. The findings will also be discussed with relevant stakeholders to support learning and inform future outbreak preparedness and response activities within the district.

Non-human subjects research using specimens/ animals collected as part of the study, or those housed in archival collections. Examples include archaeology, paleontology, botany and zoology.

Did the permission you obtained from a local authority to perform the study include an agreement on access to outputs and benefit sharing? This may include procedures to enable fair distribution of the benefits and resources arising from the research performed. Please include any details of Prior Informed Consent and Benefit Sharing Agreements obtained. These may be required by field-specific regulations, for example the Convention on Biological Diversity (CBD) and the associated Nagoya Protocol.

Not Applicable (N/A)

If the material used in your study was imported, please A) provide the year it was imported and B) indicate whether permits were obtained to import/export the materials used, C) provide details of any permits obtained. If this information is not available, please indicate this.

N/A

If you used archival specimens, please state how the material used in your study was acquired by the institute it is held in and provide details of any permits obtained for the original excavations/ sample collection. If this information is not available, please indicate this.

N/A

How was the potential cultural significance of the materials collected in your study to local communities considered in your research design? Were Indigenous peoples and/or local researchers and institutions involved with archaeological excavations / collection of specimens? If so, please provide a description of their involvement.

N/A

If your manuscript includes photographs of human remains please indicate whether authors obtained permission from descendants or affiliated cultural communities to do so.

N/A
