# Supplementary material for: Fear, stigma and Uncertainty: The short and long-term effects of Ebola on survivors, affected families, and community in Bundibugyo, Western Uganda
Source: PLoS One. 2026 Sep 8;21(9):e0355778. doi: 10.1371/journal.pone.0355778 (PMC13552781; doi:10.1371/journal.pone.0355778)
Supplement: Supplementary file 3 [file pone.0355778.s003.docx]

Shamilah Namusisi

Makerere University, Kampala, Uganda

shamilahnamusisi@gmail.com

+256 772304276

July 19^th^,2026

**Academic Editor, PLOS ONE**

Dear Editor,

**RE: Submission of Manuscript -** **Fear, Stigma and Uncertainty: The short and long-term effects of Ebola on survivors, affected families, and community in Bundibugyo, Western Uganda**

This is to bring to your attention that a resubmission of the above-mentioned manuscript after

response to reviewer comments has been made

**Response to Review#1 Comments**

Th**e** team has made additional changes based on reviewer recommednations (refer to Revised Manuscript with Track Changes);

Comments and response

1. Please better explain what an Eco-Health framework is (lines 132 and following).

**Response line 134-137:** **The Eco-health framework is an interdisciplinary approach that recognizes the interdependence of human, animal and ecosystem health and emphasizes that health outcomes are shaped by complex interactions among biological, environmental, social, cultural and economic systems.**

2. Line 161: there need to be the article before the name of the district (the)? Consider the same for the following names of places and districts. It should generally be the case in English but I acknowledge the possibility for local usage.

**Response indeed the local usage and acceptable sue of artcicles is what guided the writing here for example the district official were not aware and Bundibugyo district official were not aware**

3. Line 172. Double check grammar.

**Respinse 172-174:** **It is also surrounded by swamps, rivers such as the Humya, and the Semliki Forest, which harbors wildlife including monkeys, chimpanzees, baboons, buffaloes, and other species.**

4. Line 208 and following. Please double check grammar and flow.

**Response 213-219:** **Interviews were conducted either in English or local languages. Where necessary, an interpreter was used to facilitate the interview with respondents. The interpreter was a trained qualitative research assistant and a native of the area fluent in English, and the two local languages in Bundibugyo, Rubwisi and Rukonzo. An in-depth interview guide was used to structure and guide the interview process. All interviews were audio-recorded with the consent of participants. In addition, written notes were taken during each interview to supplement the recordings and capture key observations.**

5. Line 215. Were ethical issues were dealt with to access these data e.g. privacy, consent. If you mentioned that elsewhere e.g. below please consider whether also useful to pinpoint it here.

**Response . Yes were delt with, also included/declared in the inclusivity in global research form**

6. Line 676. Should you use EVD for the disease name? Please double check consistency of use of acronyms.

**Response this has been checked; First time as Ebola virus disease (EVD) and later EVD and again Ebola virus disease (EVD) in the concluding statement .**

7. Lines 706 and following. Please better frame the main concerns of your study such as stigma into the discussion.

**Response 714 and following , stugma has been fully addressed**

8. Line 820. Also consider adding strengths in the subheading given you are addressing the topic in the section.

**Response : the study strennth line 828 -837 and limitaions line 838-845**

9. Line 843 and following. The conclusion could be made more incisive including by briefly summarising the results of your research

**Response line 851-860: Nearly a decade after the 2007 Bundibugyo Ebola virus disease (EVD) outbreak, survivors, affected families, and communities continue to experience significant social, cultural and economic consequences. While some positive changes like improved hygiene and health-seeking behaviors were reported, persistent stigma, fear, psychosocial distress, social isolation and livelihood disruption remained the dominant long-term impacts. These findings demonstrate that recovery from EVD extends beyond the end of the outbreak. This study therefore highlights the importance of integrating social science in responding to both the short and long-term needs of communities hit by these emerging infectious diseases. Long term investment in community engagement, stigma reduction, psychosocial support, and livelihood support are essential to strengthen recovery and improve preparedness to future outbreaks.**

Thank you the continued support

Sincerely,

Shamilah Namusisi
