# Supplementary material for: Fear, stigma and Uncertainty: The short and long-term effects of Ebola on survivors, affected families, and community in Bundibugyo, Western Uganda
Source: PLoS One. 2026 Sep 8;21(9):e0355778. doi: 10.1371/journal.pone.0355778 (PMC13552781; doi:10.1371/journal.pone.0355778)
Supplement: Peer review history

# Author comment: Author response to Decision Letter 0

**DOI:** 10.1371/journal.pone.0355778.r001

25 Feb 2026

Attachment Submitted filename: Manuscript with track changes.docx

# Peer review: Decision Letter 0

**DOI:** 10.1371/journal.pone.0355778.r002

**Authors:** Jan Rychtář

28 Apr 2026

Dear Dr. Namusisi,

Thank you for submitting your manuscript to PLOS ONE. After careful consideration, we feel that it has merit but does not fully meet PLOS ONE’s publication criteria as it currently stands. Therefore, we invite you to submit a revised version of the manuscript that addresses the points raised during the review process.

Please submit your revised manuscript by Jun 12 2026 11:59PM. If you will need more time than this to complete your revisions, please reply to this message or contact the journal office at plosone@plos.org. When you're ready to submit your revision, log on to https://www.editorialmanager.com/pone/ and select the 'Submissions Needing Revision' folder to locate your manuscript file.

- A letter that responds to each point raised by the academic editor and reviewer(s). You should upload this letter as a separate file labeled 'Response to Reviewers'.
- A marked-up copy of your manuscript that highlights changes made to the original version. You should upload this as a separate file labeled 'Revised Manuscript with Track Changes'.
- An unmarked version of your revised paper without tracked changes. You should upload this as a separate file labeled 'Manuscript'.

If you would like to make changes to your financial disclosure, please include your updated statement in your cover letter. Guidelines for resubmitting your figure files are available below the reviewer comments at the end of this letter.

If applicable, we recommend that you deposit your laboratory protocols in protocols.io to enhance the reproducibility of your results. Protocols.io assigns your protocol its own identifier (DOI) so that it can be cited independently in the future. For instructions see: https://journals.plos.org/plosone/s/submission-guidelines#loc-laboratory-protocols. Additionally, PLOS ONE offers an option for publishing peer-reviewed Lab Protocol articles, which describe protocols hosted on protocols.io. Read more information on sharing protocols at https://plos.org/protocols?utm_medium=editorial-email&utm_source=authorletters&utm_campaign=protocols.

As the corresponding author, your ORCID iD is verified in the submission system and will appear in the published article. PLOS supports the use of ORCID, and we encourage all coauthors to register for an ORCID iD and use it as well. Please encourage your coauthors to verify their ORCID iD within the submission system before final acceptance, as unverified ORCID iDs will not appear in the published article. *Only*  the individual author can complete the verification step; PLOS staff *cannot*  verify ORCID iDs on behalf of authors.

We look forward to receiving your revised manuscript.

Kind regards,

Jan Rychtář

Academic Editor

PLOS One

Journal Requirements:

When submitting your revision, we need you to address these additional requirements.

1. Please ensure that your manuscript meets PLOS ONE's style requirements, including those for file naming. The PLOS ONE style templates can be found at

https://journals.plos.org/plosone/s/file?id=wjVg/PLOSOne_formatting_sample_main_body.pdf and

https://journals.plos.org/plosone/s/file?id=ba62/PLOSOne_formatting_sample_title_authors_affiliations.pdf.

2. Please include a complete copy of PLOS’ questionnaire on inclusivity in global research in your revised manuscript. Our policy for research in this area aims to improve transparency in the reporting of research performed outside of researchers’ own country or community. The policy applies to researchers who have travelled to a different country to conduct research, research with Indigenous populations or their lands, and research on cultural artefacts. The questionnaire can also be requested at the journal’s discretion for any other submissions, even if these conditions are not met.  Please find more information on the policy and a link to download a blank copy of the questionnaire here: https://journals.plos.org/plosone/s/best-practices-in-research-reporting. Please upload a completed version of your questionnaire as Supporting Information when you resubmit your manuscript.

3. Thank you for stating in your Funding Statement:

“The study was conducted with funding support from University of Minnesota as a capacity building grant under the US Agency for International Development grant (Grant number AID-OAA-A-15-00014. PI: KP, https://www.usaid.gov/work-usaid/get-grant-or-contract/opportunities-funding). There was no additional external funding received for this study. The sponsors or funders did not play any role in the study design, data collection and analysis, decision to publish, or preparation of the manuscript. “

Please provide an amended statement that declares *all* the funding or sources of support (whether external or internal to your organization) received during this study, as detailed online in our guide for authors at http://journals.plos.org/plosone/s/submit-now.  Please also include the statement “There was no additional external funding received for this study.” in your updated Funding Statement.

Please include your amended Funding Statement within your cover letter. We will change the online submission form on your behalf.

4. Please be informed that funding information should not appear in the Acknowledgments section or other areas of your manuscript. We will only publish funding information present in the Funding Statement section of the online submission form. Please remove any funding-related text from the manuscript.

5. Please ensure that you refer to Figure 1 in your text as, if accepted, production will need this reference to link the reader to the figure.

6. Please include a caption for figure 1.

7. We note that Figure 1 in your submission contain [map/satellite] images which may be copyrighted. All PLOS content is published under the Creative Commons Attribution License (CC BY 4.0), which means that the manuscript, images, and Supporting Information files will be freely available online, and any third party is permitted to access, download, copy, distribute, and use these materials in any way, even commercially, with proper attribution. For these reasons, we cannot publish previously copyrighted maps or satellite images created using proprietary data, such as Google software (Google Maps, Street View, and Earth). For more information, see our copyright guidelines: http://journals.plos.org/plosone/s/licenses-and-copyright.

We require you to either (1) present written permission from the copyright holder to publish these figures specifically under the CC BY 4.0 license, or (2) remove the figures from your submission:

a. You may seek permission from the original copyright holder of Figure 1 to publish the content specifically under the CC BY 4.0 license.

We recommend that you contact the original copyright holder with the Content Permission Form (http://journals.plos.org/plosone/s/file?id=7c09/content-permission-form.pdf) and the following text:

“I request permission for the open-access journal PLOS ONE to publish XXX under the Creative Commons Attribution License (CCAL) CC BY 4.0 (http://creativecommons.org/licenses/by/4.0/). Please be aware that this license allows unrestricted use and distribution, even commercially, by third parties. Please reply and provide explicit written permission to publish XXX under a CC BY license and complete the attached form.”

Please upload the completed Content Permission Form or other proof of granted permissions as an "Other" file with your submission.

In the figure caption of the copyrighted figure, please include the following text: “Reprinted from [ref] under a CC BY license, with permission from [name of publisher], original copyright [original copyright year].”

b. If you are unable to obtain permission from the original copyright holder to publish these figures under the CC BY 4.0 license or if the copyright holder’s requirements are incompatible with the CC BY 4.0 license, please either i) remove the figure or ii) supply a replacement figure that complies with the CC BY 4.0 license. Please check copyright information on all replacement figures and update the figure caption with source information. If applicable, please specify in the figure caption text when a figure is similar but not identical to the original image and is therefore for illustrative purposes only.

The following resources for replacing copyrighted map figures may be helpful:

USGS National Map Viewer (public domain): http://viewer.nationalmap.gov/viewer/

The Gateway to Astronaut Photography of Earth (public domain): http://eol.jsc.nasa.gov/sseop/clickmap/

Maps at the CIA (public domain): https://www.cia.gov/library/publications/the-world-factbook/index.html and https://www.cia.gov/library/publications/cia-maps-publications/index.html

NASA Earth Observatory (public domain): http://earthobservatory.nasa.gov/

Landsat: http://landsat.visibleearth.nasa.gov/

USGS EROS (Earth Resources Observatory and Science (EROS) Center) (public domain): http://eros.usgs.gov/#

Natural Earth (public domain): http://www.naturalearthdata.com/

8.Please include captions for your Supporting Information files at the end of your manuscript, and update any in-text citations to match accordingly. Please see our Supporting Information guidelines for more information: http://journals.plos.org/plosone/s/supporting-information.

9.  We note that this data set consists of interview transcripts. Can you please confirm that all participants gave consent for interview transcript to be published?

If they DID provide consent for these transcripts to be published, please also confirm that the transcripts do not contain any potentially identifying information (or let us know if the participants consented to having their personal details published and made publicly available). We consider the following details to be identifying information:

- Names, nicknames, and initials

- Age more specific than round numbers

- GPS coordinates, physical addresses, IP addresses, email addresses

- Information in small sample sizes (e.g. 40 students from X class in X year at X university)

- Specific dates (e.g. visit dates, interview dates)

- ID numbers

Or, if the participants DID NOT provide consent for these transcripts to be published:

- Provide a de-identified version of the data or excerpts of interview responses

- Provide information regarding how these transcripts can be accessed by researchers who meet the criteria for access to confidential data, including:

a) the grounds for restriction

b) the name of the ethics committee, Institutional Review Board, or third-party organization that is imposing sharing restrictions on the data

c) a non-author, institutional point of contact that is able to field data access queries, in the interest of maintaining long-term data accessibility.

d) Any relevant data set names, URLs, DOIs, etc. that an independent researcher would need in order to request your minimal data set.

For further information on sharing data that contains sensitive participant information, please see: https://journals.plos.org/plosone/s/data-availability#loc-human-research-participant-data-and-other-sensitive-data

If there are ethical, legal, or third-party restrictions upon your dataset, you must provide all of the following details (https://journals.plos.org/plosone/s/data-availability#loc-acceptable-data-access-restrictions):

a. A complete description of the dataset

b. The nature of the restrictions upon the data (ethical, legal, or owned by a third party) and the reasoning behind them

c. The full name of the body imposing the restrictions upon your dataset (ethics committee, institution, data access committee, etc)

d. If the data are owned by a third party, confirmation of whether the authors received any special privileges in accessing the data that other researchers would not have

e. Direct, non-author contact information (preferably email) for the body imposing the restrictions upon the data, to which data access requests can be sent

10. If the reviewer comments include a recommendation to cite specific previously published works, please review and evaluate these publications to determine whether they are relevant and should be cited. There is no requirement to cite these works unless the editor has indicated otherwise.

Additional Editor Comments (if provided):

The manuscript was reviewed by two reviewers, both recommend specific major revisions. Please revise accordingly carefully addressing reviewers' comments.

[Note: HTML markup is below. Please do not edit.]

Reviewers' comments:

Reviewer's Responses to Questions

**Comments to the Author**

1. Is the manuscript technically sound, and do the data support the conclusions?

Reviewer #1: Partly

Reviewer #2: Yes

**********

2. Has the statistical analysis been performed appropriately and rigorously?

Reviewer #1: N/A

Reviewer #2: Yes

**********

3. Have the authors made all data underlying the findings in their manuscript fully available?

The PLOS Data policy

Reviewer #1: Yes

Reviewer #2: Yes

**********

4. Is the manuscript presented in an intelligible fashion and written in standard English?

Reviewer #1: Yes

Reviewer #2: Yes

**********

Reviewer #1: The article addresses a topic of heighten interest in public health, namely the long-term health and social effects of Ebola virus disease. The long-term ramifications of infections are topics that have come to the fore during the Covid pandemic (including in relation to the sequelae of SARS-CoV-2 infection or Long Covid, although the present manuscript does not address post Ebola syndrome head on). However, studies addressing diseases such as EVD have received comparatively less attention in infectious disease research. The manuscript, therefore, is timely and worthy of attention.

I peer review the paper with a focus on language, flow, structure, consistency and limitations.

The paper is generally well written although it must be improved at times for better flow. I suggest to re-read it and polish the language when possible. There also appear to be possible grammar errors at times and places where the sentences are too long. Some examples below.

Please pinpoint the sample size early in the paper and if appropriate in the abstract. Please be more incisive in how you present inferences drawn from a relatively small sample.

Line 24 do you mean short-term, short-lived? Please better phrase this sentence.

Line 48. Aggregate. Unclear.

Line 49. Punctuation. Double check.

Line 50. Break the sentence for better flow.

Line 52. Complicated. Possibly choose another word or explain it better. Complicated to manage? To contain?

Line 55 and following. Break the sentences for better flow.

Line 63. Improve readability and flow.

Line 74. Grammar. Please clarify.

Line 85. I would break the sentence for additional clarity.

Line 92. I would break the sentence for additional clarity.

Line 95 and following. Please double check grammar and rephrase for additional clarity.

Line 105 and following. Please mention more clearly the sequelae (long-term health effects) of infection. I presume those not recovering went through a particularly complicated situation.

Line 127. Add bibliography including on the Covid pandemic, which obviously represents a major focus of these kinds of studies.

Line 135 and following. I would break the sentence for additional clarity.

Line 138. Add bibliography for Eco-health.

Line 148. Sculpturing. Unclear.

Line 152. In THE.. district? Double check the use of articles across the paper.

Line 154. Grammar.

Line 187. Punctuation.

How many participants broadly do you have? Please pinpoint this here already and increase the information on data sampling.

Line 195. Please break the sentence for additional clarity.

Were the 19 interviewees survivors of the disease or e.g. family members? Please provide more details.

Line 208. Grammar.

Line 228. Clarify further and improve grammar.

Line 268. Please break the sentence for additional clarity and flow.

Line 621. Do you have any information on long-term sequelae experienced by survivors and how they were treated by their community if they didn’t recover?

Line 684. Again do you know if there are people still sick with post Ebola syndrome? This is an aspect of the topic which seems underdeveloped. Potentially because at the time of the survey issues of long-term illness that came into sharper focus with Covid and Long Covid were not central to research? Address as a limitation to the study in case.

Line 751. Improve phrasing.

Line 762. Please rephrase about Covid by also addressing cases where safety measures were accepted by the community and by addressing the cases where such procedures were necessary to e.g. avoiding healthcare collapse. Please add more bibliography.

Line 783. Revise use of and.

Line 784. Among the negative experiences, do you have evidence of post Ebola syndrome in your study?

Line 787. You should describe these approaches more in detail in the methodology.

Please address your study limitations.

Reviewer #2: 1. While the study is described as a focused ethnography, several critical methodological elements are insufficiently detailed: Sample size is small (n = 19 interviews) with no clear justification of saturation; Sampling strategy (purposive + snowball) is stated but lacks detail on how participants were approached, inclusion/exclusion criteria beyond basic definitions. Also, reflexivity is missing (researcher positionality, potential bias)

My suggestion would be to explicitly describe how thematic saturation was reached; provide more detail on recruitment procedures and include reflexivity statement

2. Coding appears largely manual (Word-based) without methodological justification. No discussion of intercoder reliabolity etc. Clarify coding framework (inductive vs deductive) and how disagreements were resolved

3. The Results section contains rich quotes, but feels like storytelling rather than analysis. Integrate findings more explicitly with the conceptual framework. Highlight patterns across participants

4. Recall bias is there as data were collected ~10 years after the outbreak (2007 → 2015). Also Generalizability and scope overstated

5. Majority of males are there 70 percent or more. please broaden limitations

6. Tighten thematic structure, there are repetititve themes

**********

what does this mean?). If published, this will include your full peer review and any attached files.

If you choose “no”, your identity will remain anonymous but your review may still be made public.

**Do you want your identity to be public for this peer review?**  For information about this choice, including consent withdrawal, please see our Privacy Policy

Reviewer #1: No

Reviewer #2: No

**********

[NOTE: If reviewer comments were submitted as an attachment file, they will be attached to this email and accessible via the submission site. Please log into your account, locate the manuscript record, and check for the action link "View Attachments". If this link does not appear, there are no attachment files.]

To ensure your figures meet our technical requirements, please review our figure guidelines: https://journals.plos.org/plosone/s/figures

You may also use PLOS’s free figure tool, NAAS, to help you prepare publication quality figures: https://journals.plos.org/plosone/s/figures#loc-tools-for-figure-preparation.

NAAS will assess whether your figures meet our technical requirements by comparing each figure against our figure specifications.

# Author comment: Author response to Decision Letter 1

**DOI:** 10.1371/journal.pone.0355778.r003

29 Jun 2026

Response to Review Comments PONE-D-26-09107

The team has made specific changes to this manuscript as detailed below (refer to Revised Manuscript with Track Changes);

1. Funding statement

All fundingor sources of funding for this research/study (whether external or internal to your organization) was received from the University of Minnesota as a capacity building grant under the US Agency for International Development grant (Grant number AID-OAA-A-15-00014. PI: KP, https://www.usaid.gov/work-usaid/get-grant-or-contract/opportunities-funding). There was no additional external funding received for this study. The sponsors or funders did not play any role in the study design, data collection and analysis, decision to publish, or preparation of the manuscript.

2. Acknowledgement revised to exclude funding information to “We would like to thank Associate Professor Katey Pelican and Chery Robertson for all the support and time they have invested in the other co-authors of this work and for nurturing them into the researchers that they are today. We would also like to acknowledge the valuable support of Steven Kakooza and Onyut Howard for their assistance in compiling the data used for the map and for their contributions to the mapping process”

3. Figure 1 Caption: Figure 1:Districts in Uganda with history of Ebola Outbreaks (2000-2023). Cartographic boundaries were obtained from the United States Census Bureau TIGER Geodatabase. Data source: Ministry of Health Uganda reports. Software used is QGIS version 3.44.4-Solothurn

4. Our research dataset may be made available to qualified teams. To proceed, please contact the first author with a brief summary of the proposed research.

Reviewer #1: As recommended , general revision has been guided with a focus on language, flow, structure and , consistency, grammar , clear sentences and limitations. Please note due to major edits, lines have shifted from the original reviewed manuscript as highlighted in the revised manuscript.

Line 24 do you mean short-term, short-lived? Please better phrase this sentence. This has sentence has been revised to: Results underscored short and long term social, cultural and economic disruptions caused by the outbreak.

Line 48. Aggregate changed to merge

Line 49. Punctuation.

Line 50. Break the sentence for better flow.

Line 52. Complicated. Possibly choose another word or explain it better. Complicated to manage? To contain?

Line 55 and following. Break the sentences for better flow.

Reseponse : Line 48 to 55 revsied to: Over the last two decades, two major outbreaks of Ebola virus disease (EVD have been recorded: the West Africa outbreak ( 2013-2016), the largest and deadliest in history, recording 11,310 cases and the Eastern Democratic Republic of Congo (DRC) (2018-2020) outbreak [3,4]. The outbreak in DRC is so far believed to be the second largest and most complicated to manage due to conflict, political instability, poor infrastructure and socio-economic weaknesses causing 2,287 deaths [3,5,6]. Ebola disproportionately impacts people living in rural communities in low income countries causing disruption of economic and social activities [7,8].

Line 63. Improve readability and flow.

Response : Line 62-66: Whereas anthropologists emphasize the overall response to the outbreak should be guided by basic principle values such as human decency, dignity, and respect[14], the outbreak in West Africa demonstrated that delays in the acknowledgment of the outbreak, unclear response roles and underdeveloped health care systems aggravated the overall impact of the outbreak on affected communities[14,15].

Line 74. Grammar. Please clarify.

Reseponse :Line 74: However, this alone cannot comprehensively explain the devastating consequences the disease in the lives of those diagnosed with EVD and their families [19,20].

Line 85. I would break the sentence for additional clarity.

Response : Line 85-87: EVD survivors often returned to their communities following -recovery to find their homes and possessions destroyed and in some cases, their spouses had abandoned them due to fear and stigma [26].

Line 92. I would break the sentence for additional clarity.

Line 95 and following. Please double check grammar and rephrase for additional clarity.

Response: Line 94- 97 : Uganda has and continues to experience outbreaks of Ebola virus disease (EVD) and the most recent (May 2026, second outbreak due to Bundibugyo ebolavirus) is ongoing. Previous outbreaks include the 2022 outbreak in Mubende district that spread to other neighboring districts [29,30]. The country’s first and largest outbreak occurred in the Northern district of Gulu in 2000 [31,32].

Line 105 and following. Please mention more clearly the sequelae (long-term health effects) of infection. I presume those not recovering went through a particularly complicated situation.

Response 107-110 : Ebola survivors often experience long-term physical health problems such as persistent joint pain, headaches, vision impairment, and fatigue, years after infection[37,40,41]. Additionally, they are at higher risk of mental health challenges such as anxiety, panic episodes, sleep disturbances and post-traumatic stress[41].

Line 127. Add bibliography including on the Covid pandemic, which obviously represents a major focus of these kinds of studies.

Response: we have incorporated aspects of COVID-19 in the discusions

Line 135 and following. I would break the sentence for additional clarity. Deleted some space and this has caused changes

Reseponse : We used an Eco-health approach integrated with perspectives on disease-related stigma to design the study and interpret reported individual, familial, and community experiences on EVD survivorship in the ten years since the Bundibugyo outbreak.

Line 138. Add bibliography for Eco-health reference number 46

Line 148. Sculpturing. Unclear. Changed to Line 145-147: In recognition of the transdisciplinary nature of this work, critical realist ontology and epistemology was employed to help derive an explanatory framework of the forces underpinning stigma.

Line 152. In THE.. district? Double check the use of articles across the paper.

Line 154. Grammar. Study area: This study was conducted in Bundibugyo district and data were collected over a period of three months from October to December 2015. The district has a population of 264,778 people and area coverage of 850.0 km² Area[46], lies in western Uganda bordering the DRC and is the only district west of the Rwenzori Mountains. It is bordered by Ntoroko District to the northeast, Kibaale District to the east, Kabarole District to the south, and the DRC to the west and north. The district headquarters at Bundibugyo town council are located approximately 32 kilometers (20 miles), by road, west of Fort Portal

Response: Line 161-164: Study area: This study was conducted in Bundibugyo district in western Uganda. It is the only district west of the Rwenzori Mountains covering 850.0 km² and home to a population of 264,778 people [49]. Bundibugyo district borders the DRC in the west and north, Ntoroko district to the northeast, Kibaale district to the east and Kabarole district to the south. The district is relatively isolated from the rest of Uganda and its inhabitants, and their customs are more similar to those of eastern DRC than the rest of Uganda

Line 187. Punctuation. How many participants broadly do you have? Please pinpoint this here already and increase the information on data sampling. Were the 19 interviewees survivors of the disease or e.g. family members? Please provide more details. Line 185-192: Participant sampling: Initially, purposive sampling and subsequently snowball sampling techniques were used to identify interview participants. All study participants were 18 years and older and included survivors, affected families and community members that were residents in the area at the time of the outbreak. Survivors were adults who self-reported laboratory confirmed EVD infection, and who were hospitalized and discharged after laboratory confirmed negative status. Affected families were those who had a member suffer from EVD regardless of whether they survived or succumbed to the disease. The community refers to residents of the study area who were present at the time of the outbreak.

Response : Line 191-206: Participant sampling: A total of 19 respondents were included in the study. Participants were categorized as survivors, affected family, and general community members. Survivors were adults who self-reported laboratory confirmed EVD infection, and who were hospitalized and discharged after laboratory confirmed negative status. Affected families were defined as households that had experienced at least one member diagnosed with EVD, regardless of whether the individual survived or died. Community members were defined as residents of the study area who were physically present during the outbreak period and thus able to provide contextual community-level perspectives. The study applied a combination of two non-probability sampling techniques. First, purposive sampling was employed to identify respondents through review of health records at Bundibugyo district hospital and information provided by health workers involved in the outbreak response. This approach ensured the inclusion of individuals with relevant lived experiences of infection, care, loss, and recovery during the outbreak. Subsequently, snowball sampling was used to recruit additional eligible participants. Participants initially identified through purposive sampling referred the research team to other potential respondents within their social or community networks. This approach was particularly useful in the post-outbreak settings where stigma, relocation, or incomplete records could hinder direct participant identification and recruitment.

Line 195-199 Please break the sentence for additional clarity. In-depth interviews: Interviews were conducted either in English or local languages with the aid of an interpreter who was a trained qualitative research assistant and a native of the area, fluent in Rubwisi and Rukonzo (the two major local languages in Bundibugyo) and highly proficient in English. An in-depth interview guide provided the interview framework. All interviews were audio recorded with participant consent and written notes were taken during the interviews. A total of 19 interviews were conducted.

Response : Line 207-213: In-depth interviews: Interviews were conducted either in English or local languages. Where necessary, an interpreter was used to facilitate the interview with respondents. The interpreter was a trained qualitative research assistant and a native of the area fluent in English, and two local languages in the area, Rubwisi and Rukonzo. An in-depth interview guide was used to structure and guide the interviews. All interviews were audio-recorded with the consent of participants. In addition, written notes were taken during each interview to supplement the recordings and capture key observations

Line 207 and 208. Grammar. Data management: Verbatim transcription of the audio files was performed and the interviews that were conducted in local languages translated into English.

Response : 229-230: Data management: Verbatim transcription of the audio recordings was undertaken, and interviews conducted in local languages were translated into English

Line 268. Please break the sentence for additional clarity and flow. This has been incorperated under the whole section of data collection

Line 621. Do you have any information on long-term sequelae experienced by survivors and how they were treated by their community if they didn’t recover?

Response : Yes the long term sequelae experiences by suvivors have been highlighted under section. However its important to note that survivors were detained in the treatment unit recovery and after they tested negative for Ebola virus

Line 684. Again do you know if there are people still sick with post Ebola syndrome? This is an aspect of the topic which seems underdeveloped. Potentially because at the time of the survey issues of long-term illness that came into sharper focus with Covid and Long Covid were not central to research? Address as a limitation to the study in case.

Line 751. Improve phrasing.

Line 762. Please rephrase about Covid by also addressing cases where safety measures were accepted by the community and by addressing the cases where such procedures were necessary to e.g. avoiding healthcare collapse. Please add more bibliography.

Respnse: :Lessons from COVID-19 further indicate that, heavy-handed bans risk numerous unintended consequences and tend to fail if they lack legitimacy or clash with people’s values [64].

Line 783. Revise use of and. Revsied line 787

Line 784. Among the negative experiences, do you have evidence of post Ebola syndrome in your study?

Response: Line 616-619. Chronic health conditions: Survivors experienced a recovery trajectory characterized by both psychological and physical sequelae, further complicated by the trauma they faced while in the isolation ward. Commonly reported symptoms include generalized body weakness, headache, hypertension, pneumonia, diabetes, and impaired vision.

Line 787. You should describe these approaches more in detail in the methodology.

Please address your study limitations.

Response : Line 831-842 Study Limitaions: Despite these strengths, several limitations should be acknowledged. First, because data were collected many years after the outbreak, the possibility of recall bias cannot be entirely excluded. Participants may have experienced difficulty accurately recalling certain events, perceptions, or experiences related to the outbreak. However, several measures were taken to mitigate this risk, including the triangulation of findings across multiple data sources and participant groups. In addition, the Ebola outbreak represented a highly significant and disruptive life event for affected individuals and communities, making key experiences and memories more likely to be retained over time. While recall bias remains a consideration, we believe its influence on the overall findings was minimized through these methodological approaches. Finally, as with many qualitative and context-specific studies, the findings should be interpreted within the social and geographic context in which the research was conducted, although they may offer important insights for understanding the long-term consequences of epidemics in similar settings.

Reviewer #2: 1. While the study is described as a focused ethnography, several critical methodological elements are insufficiently detailed: Sample size is small (n = 19 interviews) and how saturation was attained

Sampling strategy (purposive + snowball) is stated but lacks detail on how participants were approached, inclusion/exclusion criteria beyond basic definitions. Also, reflexivity is missing (researcher positionality, potential bias). This has been highlighted above

2. Coding appears largely manual (Word-based) without methodological justification. No discussion of intercoder reliabolity etc. Clarify coding framework (inductive vs deductive) and how disagreements were resolved. Revised

3. The Results section contains rich quotes, but feels like storytelling rather than analysis. Integrate findings more explicitly with the conceptual framework. Highlight patterns across participants

4. Recall bias is there as data were collected ~10 years after the outbreak (2007 → 2015). Also Generalizability and scope overstated.

5. Majority of males are there 70 percent or more. please broaden limitations

6. Tighten thematic structure, there are repetititve themes

Response to Reviewer #2

We have also expanded the description of the sampling strategy under reviwer #1 comments and Participants were initially purposively selected based on their direct experience with the Ebola outbreak and its social consequences. Inclusion criteria required participants to be adults (≥18 years) residing in the study area during or after the outbreak and possessing relevant experiences related to Ebola-related stigma. Individuals unable to

# Peer review: Decision Letter 1

**DOI:** 10.1371/journal.pone.0355778.r004

**Authors:** Jan Rychtář

10 Jul 2026

Dear Dr. Namusisi,

Thank you for submitting your manuscript to PLOS ONE. After careful consideration, we feel that it has merit but does not fully meet PLOS ONE’s publication criteria as it currently stands. Therefore, we invite you to submit a revised version of the manuscript that addresses the points raised during the review process.

==============================

Only a small list of minor revisions remains before the manuscript is acceptable

==============================

Please submit your revised manuscript by Aug 24 2026 11:59PM. If you will need more time than this to complete your revisions, please reply to this message or contact the journal office at plosone@plos.org. When you're ready to submit your revision, log on to https://www.editorialmanager.com/pone/ and select the 'Submissions Needing Revision' folder to locate your manuscript file.

- A letter that responds to each point raised by the academic editor and reviewer(s). You should upload this letter as a separate file labeled 'Response to Reviewers'.
- A marked-up copy of your manuscript that highlights changes made to the original version. You should upload this as a separate file labeled 'Revised Manuscript with Track Changes'.
- An unmarked version of your revised paper without tracked changes. You should upload this as a separate file labeled 'Manuscript'.

If you would like to make changes to your financial disclosure, please include your updated statement in your cover letter. Guidelines for resubmitting your figure files are available below the reviewer comments at the end of this letter.

If applicable, we recommend that you deposit your laboratory protocols in protocols.io to enhance the reproducibility of your results. Protocols.io assigns your protocol its own identifier (DOI) so that it can be cited independently in the future. For instructions see: https://journals.plos.org/plosone/s/submission-guidelines#loc-laboratory-protocols. Additionally, PLOS ONE offers an option for publishing peer-reviewed Lab Protocol articles, which describe protocols hosted on protocols.io. Read more information on sharing protocols at https://plos.org/protocols?utm_medium=editorial-email&utm_source=authorletters&utm_campaign=protocols.

As the corresponding author, your ORCID iD is verified in the submission system and will appear in the published article. PLOS supports the use of ORCID, and we encourage all coauthors to register for an ORCID iD and use it as well. Please encourage your coauthors to verify their ORCID iD within the submission system before final acceptance, as unverified ORCID iDs will not appear in the published article. *Only*  the individual author can complete the verification step; PLOS staff *cannot*  verify ORCID iDs on behalf of authors.

We look forward to receiving your revised manuscript.

Kind regards,

Jan Rychtář

Academic Editor

PLOS One

Journal Requirements:

If the reviewer comments include a recommendation to cite specific previously published works, please review and evaluate these publications to determine whether they are relevant and should be cited. There is no requirement to cite these works unless the editor has indicated otherwise.

Please review your reference list to ensure that it is complete and correct. If you have cited papers that have been retracted, please include the rationale for doing so in the manuscript text, or remove these references and replace them with relevant current references. Any changes to the reference list should be mentioned in the rebuttal letter that accompanies your revised manuscript. If you need to cite a retracted article, indicate the article’s retracted status in the References list and also include a citation and full reference for the retraction notice.

Additional Editor Comments (if provided):

This version largely addresses the previous round of comments and only a small list of minor revisions remains in order for the manuscript to be acceptable.

[Note: HTML markup is below. Please do not edit.]

Reviewers' comments:

Reviewer's Responses to Questions

**Comments to the Author**

Reviewer #1: All comments have been addressed

**********

2. Is the manuscript technically sound, and do the data support the conclusions?

Reviewer #1: Yes

**********

3. Has the statistical analysis been performed appropriately and rigorously?

Reviewer #1: N/A

**********

4. Have the authors made all data underlying the findings in their manuscript fully available?

The PLOS Data policy

Reviewer #1: Yes

**********

5. Is the manuscript presented in an intelligible fashion and written in standard English?

Reviewer #1: Yes

**********

Reviewer #1: I thank the authors for addressing my comments and reviewing extensively the manuscript, which greatly improved the article.

A few more comments for additional improvements. Please revise the manuscript one more time. Some sentences could be shortened and improved at times. Please double check the spacing across the manuscript (I see lack of spaces at times). The paper is however generally very well written and grammatically correct.

Please better explain what an Eco-Health framework is (lines 132 and following).

Line 161: there need to be the article before the name of the district (the)? Consider the same for the following names of places and districts. It should generally be the case in English but I acknowledge the possibility for local usage.

Line 172. Double check grammar.

Line 208 and following. Please double check grammar and flow.

Line 215. Were ethical issues were dealt with to access these data e.g. privacy, consent. If you mentioned that elsewhere e.g. below please consider whether also useful to pinpoint it here.

Line 676. Should you use EVD for the disease name? Please double check consistency of use of acronyms.

Lines 706 and following. Please better frame the main concerns of your study such as stigma into the discussion.

Line 820. Also consider adding strengths in the subheading given you are addressing the topic in the section.

Line 843 and following. The conclusion could be made more incisive including by briefly summarising the results of your research.

**********

what does this mean?). If published, this will include your full peer review and any attached files.

If you choose “no”, your identity will remain anonymous but your review may still be made public.

**Do you want your identity to be public for this peer review?**  For information about this choice, including consent withdrawal, please see our Privacy Policy

Reviewer #1: No

**********

[NOTE: If reviewer comments were submitted as an attachment file, they will be attached to this email and accessible via the submission site. Please log into your account, locate the manuscript record, and check for the action link "View Attachments". If this link does not appear, there are no attachment files.]

To ensure your figures meet our technical requirements, please review our figure guidelines: https://journals.plos.org/plosone/s/figures

You may also use PLOS’s free figure tool, NAAS, to help you prepare publication quality figures: https://journals.plos.org/plosone/s/figures#loc-tools-for-figure-preparation.

NAAS will assess whether your figures meet our technical requirements by comparing each figure against our figure specifications.

# Author comment: Author response to Decision Letter 2

**DOI:** 10.1371/journal.pone.0355778.r005

22 Jul 2026

Response to Review#1 Comments

The team has made additional changes based on reviewer recommednations (refer to Revised Manuscript with Track Changes);

Comments and response

1. Please better explain what an Eco-Health framework is (lines 132 and following).

Response line 134-137: The Eco-health framework is an interdisciplinary approach that recognizes the interdependence of human, animal and ecosystem health and emphasizes that health outcomes are shaped by complex interactions among biological, environmental, social, cultural and economic systems.

2. Line 161: there need to be the article before the name of the district (the)? Consider the same for the following names of places and districts. It should generally be the case in English but I acknowledge the possibility for local usage.

Response indeed the local usage and acceptable sue of artcicles is what guided the writing here for example the district official were not aware and Bundibugyo district official were not aware

3. Line 172. Double check grammar.

Respinse 172-174: It is also surrounded by swamps, rivers such as the Humya, and the Semliki Forest, which harbors wildlife including monkeys, chimpanzees, baboons, buffaloes, and other species.

4. Line 208 and following. Please double check grammar and flow.

Response 213-219: Interviews were conducted either in English or local languages. Where necessary, an interpreter was used to facilitate the interview with respondents. The interpreter was a trained qualitative research assistant and a native of the area fluent in English, and the two local languages in Bundibugyo, Rubwisi and Rukonzo. An in-depth interview guide was used to structure and guide the interview process. All interviews were audio-recorded with the consent of participants. In addition, written notes were taken during each interview to supplement the recordings and capture key observations.

5. Line 215. Were ethical issues were dealt with to access these data e.g. privacy, consent. If you mentioned that elsewhere e.g. below please consider whether also useful to pinpoint it here.

Response . Yes were delt with, also included/declared in the inclusivity in global research form

6. Line 676. Should you use EVD for the disease name? Please double check consistency of use of acronyms.

Response this has been checked; First time as Ebola virus disease (EVD) and later EVD and again Ebola virus disease (EVD) in the concluding statement .

7. Lines 706 and following. Please better frame the main concerns of your study such as stigma into the discussion.

Response 714 and following , stugma has been fully addressed

8. Line 820. Also consider adding strengths in the subheading given you are addressing the topic in the section.

Response : the study strennth line 828 -837 and limitaions line 838-845

9. Line 843 and following. The conclusion could be made more incisive including by briefly summarising the results of your research

Response line 851-860: Nearly a decade after the 2007 Bundibugyo Ebola virus disease (EVD) outbreak, survivors, affected families, and communities continue to experience significant social, cultural and economic consequences. While some positive changes like improved hygiene and health-seeking behaviors were reported, persistent stigma, fear, psychosocial distress, social isolation and livelihood disruption remained the dominant long-term impacts. These findings demonstrate that recovery from EVD extends beyond the end of the outbreak. This study therefore highlights the importance of integrating social science in responding to both the short and long-term needs of communities hit by these emerging infectious diseases. Long term investment in community engagement, stigma reduction, psychosocial support, and livelihood support are essential to strengthen recovery and improve preparedness to future outbreaks.

Attachment Submitted filename: Response to Reviewers.docx

# Editor report: Decision Letter 2

**DOI:** 10.1371/journal.pone.0355778.r006

**Authors:** Jan Rychtář

26 Jul 2026

Fear, Stigma and Uncertainty: The short and long-term effects of Ebola on survivors, affected families, and community in Bundibugyo, Western Uganda

PONE-D-26-09107R2

Dear Dr. Namusisi,

We’re pleased to inform you that your manuscript has been judged scientifically suitable for publication and will be formally accepted for publication once it meets all outstanding technical requirements.

Within one week, you’ll receive an e-mail detailing the required amendments. When these have been addressed, you’ll receive a formal acceptance letter and your manuscript will be scheduled for publication.

An invoice will be generated when your article is formally accepted. Please note, if your institution has a publishing partnership with PLOS and your article meets the relevant criteria, all or part of your publication costs will be covered. Please make sure your user information is up-to-date by logging into Editorial Manager at Editorial Manager® and clicking the ‘Update My Information' link at the top of the page. For questions related to billing, please contact billing support.

If your institution or institutions have a press office, please notify them about your upcoming paper to help maximize its impact. If they’ll be preparing press materials, please inform our press team as soon as possible -- no later than 48 hours after receiving the formal acceptance. Your manuscript will remain under strict press embargo until 2 pm Eastern Time on the date of publication. For more information, please contact onepress@plos.org.

Kind regards,

Jan Rychtář

Academic Editor

PLOS One

Additional Editor Comments (optional):

The authors addressed the comments adequately

Reviewers' comments:

# Editor report: Acceptance letter

**DOI:** 10.1371/journal.pone.0355778.r007

**Authors:** Jan Rychtář

PONE-D-26-09107R2

PLOS One

Dear Dr. Namusisi,

I'm pleased to inform you that your manuscript has been deemed suitable for publication in PLOS One. Congratulations! Your manuscript is now being handed over to our production team.

At this stage, our production department will prepare your paper for publication. This includes ensuring the following:

* All references, tables, and figures are properly cited

* All relevant supporting information is included in the manuscript submission,

* There are no issues that prevent the paper from being properly typeset

You will receive further instructions from the production team, including instructions on how to review your proof when it is ready. Please keep in mind that we are working through a large volume of accepted articles, so please give us a few days to review your paper and let you know the next and final steps.

Lastly, if your institution or institutions have a press office, please let them know about your upcoming paper now to help maximize its impact. If they'll be preparing press materials, please inform our press team within the next 48 hours. Your manuscript will remain under strict press embargo until 2 pm Eastern Time on the date of publication. For more information, please contact onepress@plos.org.

You will receive an invoice from PLOS for your publication fee after your manuscript has reached the completed accept phase. If you receive an email requesting payment before acceptance or for any other service, this may be a phishing scheme. Learn how to identify phishing emails and protect your accounts at https://explore.plos.org/phishing.

If we can help with anything else, please email us at customercare@plos.org.

Thank you for submitting your work to PLOS One and supporting open access.

Kind regards,

PLOS One Editorial Office Staff

on behalf of

Dr. Jan Rychtář

Academic Editor

PLOS One
